# Epigenetic variation during the adult lifespan: cross-sectional and longitudinal data on monozygotic twin pairs

**DOI:** 10.1111/j.1474-9726.2012.00835.x

**Published:** 2012-08

**Authors:** Rudolf P Talens, Kaare Christensen, Hein Putter, Gonneke Willemsen, Lene Christiansen, Dennis Kremer, H Eka D Suchiman, P Eline Slagboom, Dorret I Boomsma, Bastiaan T Heijmans

**Affiliations:** 1 Department of Molecular Epidemiology, Leiden University Medical CenterLeiden, The Netherlands; 2 The Danish Aging Research Center and The Danish Twin Registry, University of Southern DenmarkOdense C, Denmark; 3 Department of Clinical Genetics, Odense University HospitalOdense C, Denmark; 4 Department of Clinical Biochemistry and Pharmacology, Odense University HospitalOdense C, Denmark; 5 Department of Medical Statistics and Bioinformatics, Leiden University Medical CenterLeiden, The Netherlands; 6 Department of Biological Psychology, VU University AmsterdamAmsterdam, The Netherlands; 7 Netherlands Consortium for Healthy AgeingLeiden, The Netherlands

**Keywords:** epigenetics, aging, MZ twin design, full adult lifespan, DNA methylation, stochastic variation

## Abstract

The accumulation of epigenetic changes was proposed to contribute to the age-related increase in the risk of most common diseases. In this study on 230 monozygotic twin pairs (MZ pairs), aged 18–89 years, we investigated the occurrence of epigenetic changes over the adult lifespan. Using mass spectrometry, we investigated variation in global (LINE1) DNA methylation and in DNA methylation at *INS*, *KCNQ1OT1*, *IGF2*, *GNASAS*, *ABCA1*, *LEP*, and *CRH*, candidate loci for common diseases. Except for *KCNQ1OT1*, interindividual variation in locus-specific DNA methylation was larger in old individuals than in young individuals, ranging from 1.2-fold larger at *ABCA1* (*P* = 0.010) to 1.6-fold larger at *INS* (*P* = 3.7 × 10^−07^). Similarly, there was more within-MZ-pair discordance in old as compared with young MZ pairs, except for *GNASAS*, ranging from an 8% increase in discordance each decade at *CRH* (*P* = 8.9 × 10^−06^) to a 16% increase each decade at *LEP* (*P* = 2.0 × 10^−08^). Still, old MZ pairs with strikingly similar DNA methylation were also observed at these loci. After 10-year follow-up in elderly twins, the variation in DNA methylation showed a similar pattern of change as observed cross-sectionally. The age-related increase in methylation variation was generally attributable to unique environmental factors, except for CRH, for which familial factors may play a more important role. In conclusion, sustained epigenetic differences arise from early adulthood to old age and contribute to an increasing discordance of MZ twins during aging.

## Introduction

The risk of most common diseases increases with age. A lifetime of accumulated epigenetic changes was proposed to contribute to the development of such diseases (Bjornsson *et al.*, 2004). Epigenetic mechanisms determine the expression potential of genes without changing the DNA sequence (Jaenisch & Bird, 2003). The molecular basis includes the methylation of cytosines in CpG dinucleotides, which, together with histone modifications, noncoding RNAs, and localization, influence the accessibility of a genomic locus to the transcriptional machinery (Bernstein *et al.*, 2007; Cedar & Bergman, 2009). DNA methylation can be measured on DNA samples that are commonly available in biobanks (Talens *et al.*, 2010).

Various studies have investigated whether DNA methylation can change with increasing calendar age. A cross-sectional study of limited sample size reported the genome-wide absence of changes in mean DNA methylation between young (26 years) and old (68 years) individuals (Eckhardt *et al.*, 2006). However, cross-sectional studies that focus on changes in mean DNA methylation can only detect age-related changes that are in the same direction for most individuals. A cross-sectional study that focussed on DNA methylation at the *COX7A1* locus reported greater interindividual variation in 20 elderly individuals (> 60 years old) compared with 20 young individuals (< 30 years old; Ronn *et al.*, 2008), indicating that DNA methylation can indeed change with age in a direction that differs per individual.

Longitudinal studies are even better suited to investigate this type of age-related methylation changes, even though most of them rarely span more than a period of two decades. A study on global DNA methylation, a measure of the average methylation level of (a representative portion of) CpG sites across the genome, observed changes with a direction that was individual specific in whole-blood samples of 111 individuals (59–86 years) followed over 11 years and 127 individuals (5–72 years) followed over 16 years (Bjornsson *et al.*, 2008). Yet, two smaller studies with 10–20 years of follow-up demonstrated that at specific genomic loci, DNA methylation in blood and buccal swab samples can remain remarkably stable (Feinberg *et al.*, 2010; Talens *et al.*, 2010).

A particularly powerful design to investigate the accumulation of epigenetic changes with age is studies of monozygotic twins (Bell & Spector, 2011). MZ co-twins have the same age and a virtually identical genotype, thus controlling for their effect on DNA methylation (Heijmans *et al.*, 2007; Bell *et al.*, 2011), while they may differ in their lifetime exposure to environmental factors and can develop small phenotypic differences with age (Martin *et al.*, 1997). To our knowledge, only one study has as yet adopted this design to study age-related changes into adulthood. This study on 40 MZ pairs aged 3–74 years reported that older MZ pairs (> 28 years old) showed larger within-pair epigenetic differences than younger MZ pairs (< 28 years old) in total DNA methylation and total histone acetylation levels. Although the functional relevance of such measures is uncertain, analysis on smaller subsets of MZ twins indicated similar trends at sites throughout the genome, most of which were repetitive sequences, but also included singly copy genes (Fraga *et al.*, 2005). Also, it remains unclear at what age such changes start to arise in the population and at what rate they subsequently progress with increasing age.

The studies performed thus far were generally relatively small, focused on measures of average methylation of the genome, and/or could investigate limited periods of the adult lifespan only. Here, we report on age-related changes in locus-specific DNA methylation in a combined cross-sectional and longitudinal study on 460 individuals comprising 230 MZ pairs aged 18–89 years. The long age range investigated allowed us to study whether epigenetic changes accumulate linearly, exponentially, or in bursts during the full adult lifespan. Furthermore, we evaluated the influence of familial versus individual factors on the age-related increase in discordance. We assessed both global DNA methylation with the LINE1 assay (Wang *et al.*, 2010) and locus-specific DNA methylation close to genes implicated in various age-related diseases, namely the nonimprinted loci *LEP*, *ABCA1*, and *CRH* and the imprinted loci *IGF2*, *INS* (alternate symbol *INSIGF*), *KCNQ1OT1* (alternate symbol *KVDMR*), and *GNASAS* (alternate symbol *NESPAS*). These loci were selected on the basis of their previously shown features of epigenetic regulation as observed in human, animal, or cell culture experiments (Mitsuya *et al.*, 1999; Melzner *et al.*, 2002; Cui *et al.*, 2003; Probst *et al.*, 2004; McGill *et al.*, 2006; Williamson *et al.*, 2006; Kuroda *et al.*, 2009; Table 1).

**Table 1:** Characteristics of the methylation assays and their corresponding loci

Locus (alias)†	Candidate for	Assay on	Literature‡	CpG island	Imprinted	CpGs§ Units	Sites
*ABCA1*¶	Cholesterol transport	Proximal promoter	± (Probst *et al.*, 2004)	+		10	18
*CRH*††	Stress response	Proximal promoter	+ (McGill *et al.*, 2006)			7	7
*GNASAS* (*NESPAS*)††	cAMP-dependent pathway	Antisense promoter	+ (Williamson *et al.*, 2006)		+	10	18
*IGF2*††	Growth and insulin signaling	DMR	+ (Cui *et al.*, 2003)		+	4	5
*IGF2*-pter	Growth and insulin signaling	pter of DMR	+ (Cui *et al.*, 2003)		+	5	5
*IGF2-*qter	Growth and insulin signaling	qter of DMR	+ (Cui *et al.*, 2003)		+	7	8
*INS* (*INSIGF*)	Glucose metabolism (placental development)	Proximal promoter	+ (Kuroda *et al.*, 2009)		+	5	5
*KCNQ1OT1* (*KVDMR*)††	Development, growth, and metabolism	Antisense promoter	+ (Mitsuya *et al.*, 1999)	+	+	11	15
*LEP*††	Metabolism	Proximal promoter	+ (Melzner *et al.*, 2002)	+		7	10
LINE1††	Proxy for global DNA methylation	5′ promoter (conserved)	± (Wang *et al.*, 2010)			8	11

† Loci are given in alphabetical order.

‡ CpG-site methylation previously reported to associate with gene expression (marked by +), ± means that this association is hinted at.

§ Amount of CpG units and CpG sites as measured in this study.

¶ For ABCA1, only the methylated CpG sites at the 5′ end of the assay are used in analyses (Tobi *et al.*, 2009).

†† Methylation of these assays was investigated over the full adult lifespan and over a 10-year follow-up period in elderly twins.

## Results

### DNA methylation in young and old MZ twins

#### Means and interindividual variation

Global DNA methylation and methylation status at nine specific loci were compared between young twins (*n* = 132 individuals) and old twins (*n* = 134 individuals). Old individuals had slightly lower mean global DNA methylation than young individuals (Table 2). Larger differences were observed at specific loci. Old individuals had lower mean DNA methylation at five of nine loci (*INS*, *KCNQ1OT1*, and the three adjacent *IGF2* loci; Table 2) and showed higher mean DNA methylation at three loci (*LEP*, *ABCA1*, and *GNASAS*). No difference was observed for *CRH*.

**Table 2:** Average DNA methylation in young and old individuals

	Mean % methylation (SD)		Difference of mean, % methylation		Fold change in variation	
			
Locus	Young	Old	Old − Young (SE)	*P*_Mean_	SD_Old_/SD_Young_	*P*_variation_
Global	61.3 (1.2)	60.9 (1.8)	−0.4 (0.19)	0.033	1.5	2.3 × 10^−05^
*KCNQ1OT1*	31.2 (2.3)	29.1 (2.3)	−2.1 (0.28)	2.4 × 10^−12^	1.0	0.999
*GNASAS*	47.3 (3.2)	52.7 (4.4)	+5.4 (0.46)	4.1 × 10^−25^	1.3	0.002
*ABCA1*	34.9 (7.8)	38.5 (9.6)	+3.6 (1.04)	6.2 × 10^−04^	1.2	0.010
*INS*	81.2 (2.3)	77.6 (3.9)	−3.6 (0.38)	9.8 × 10^−18^	1.6	3.7 × 10^−07^
*IGF2DMR*	54.7 (3.8)	51.2 (4.9)	−3.5 (0.52)	2.8 × 10^−10^	1.3	0.002
*IGF2_qter*	70.9 (2.8)	68.5 (5.4)	−2.4 (0.52)	1.3 × 10^−05^	1.9	4.6 × 10^−07^
*IGF2_pter*	45.7 (3.0)	42.1 (4.4)	−3.6 (0.45)	6.5 × 10^−14^	1.5	3.5 × 10^−05^
*LEP*	27.6 (4.2)	32.2 (6.4)	+4.5 (0.65)	3.6 × 10^−11^	1.5	1.3 × 10^−05^
*CRH*	63.4 (4.3)	63.7 (6.6)	+0.3 (0.67)	0.671	1.5	2.2 × 10^−06^

The interindividual variation in global DNA methylation expressed as the standard deviation (SD) was small in both age groups (SD_young_ = 1.2% and SD_old_ = 1.8%; Table 2). At specific loci, it ranged from small in both age groups at *KCNQ1OT1* (SD = 2.3% in both age groups) to large in both age groups at *ABCA1* (SD_young_ = 7.8% and SD_old_ = 9.6%; Table 2). With the exception of *KCNQ1OT1*, methylation variation was always larger in old individuals than in young individuals, irrespective of age-related differences in mean DNA methylation. The SD of global DNA methylation was 1.5-fold larger in old individuals (*P* = 2.3 × 10^−05^). At specific loci, the age-related difference ranged from 1.2-fold larger SD at *ABCA1* (*P* = 0.010) to 1.6-fold larger SD at *INS* (*P* = 3.7 × 10^−07^; Table 2).

#### Within-pair discordance

**Fig. 1:**
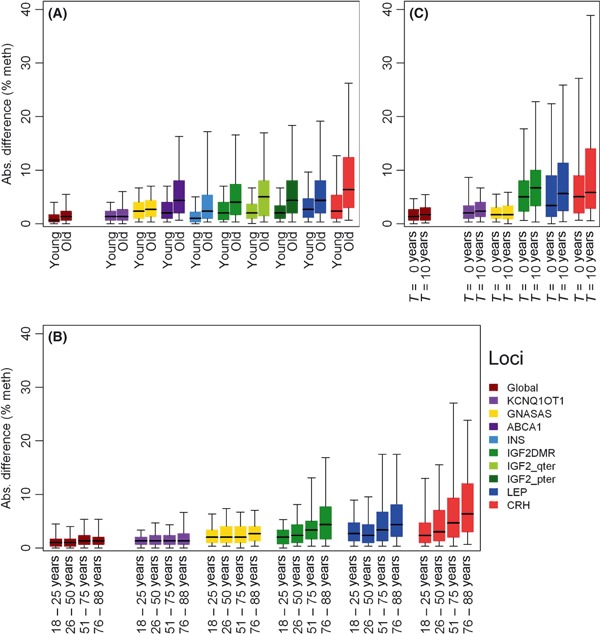
Increase in within-pair methylation discordance with age. Absolute within-pair difference in percent DNA methylation for global DNA methylation and for specific loci plotted for (A) Young MZ twins (below 30 years old; *n* = 66 pairs) vs. old MZ twins (above 74 years old; *n* = 67 pairs). (B) The full age range divided by the four stages of adult life; from left to right: up to 25 years of age (early adulthood, *n* = 30 pairs), 26 up to 50 years of age (early middle age, *n* = 78 pairs), 51 up to 75 years of age (late middle age, *n* = 56 pairs), and above 76 years of age (old age, *n* = 54 pairs). (C) 19 elderly MZ pairs (73–82 years old), after 10 years of follow-up (83–92 years old). The bars show the interquartile range, the thick line in the center of the bar shows the median, and the whiskers show the 5th (bottom whisker) and 95th (top whisker) percentiles.

The extent of within-pair methylation discordance was also compared between the young and old twins. Similar to the interindividual variation, a small within-pair discordance in global methylation was observed in both age groups. At specific loci, it ranged from small in both age groups at *KCNQ1OT1* to large in both age groups at *CRH*. Furthermore, the absolute within-pair discordance in old MZ pairs was always greater than in young MZ pairs (Fig. 1A). Notwithstanding this overall increase, the old age group still contained pairs who had strikingly similar DNA methylation. With the exception of *GNASAS*, the SD of the within-pair differences, quantifying group discordance, was significantly higher in old as compared with young MZ pairs (Table 3). For global DNA methylation, methylation discordance in the old MZ pairs was almost double that of the young MZ pairs (*P* = 9.8 × 10^−05^). At specific loci, the increase in discordance ranged from 1.4-fold greater in old MZ pairs at *KCNQ1OT1* (*P* = 0.005) to 2.7-fold greater at *ABCA1* (*P* = 3.8 × 10^−07^).

**Table 3:** Variation of within-MZ-pair methylation difference in young and old MZ pairs

	Estimate of variation as SD (% methylation)†		Fold change in variation	
		
Locus	Young MZ pairs	Old MZ pairs	Old/young	*P*_variation_
Global	1.1	2.1	1.9	9.8 × 10^−05^
*KCNQ1OT1*	1.9	2.6	1.4	0.005
*GNASAS*	3.1	3.4	1.1	0.193
*ABCA1*	2.8	7.7	2.7	3.8 × 10^−07^
*INS*	2.1	4.6	2.2	2.3 × 10^−06^
*IGF2DMR*	2.6	4.9	1.9	2.1 × 10^−05^
*IGF2_qter*	2.4	5.9	2.4	9.7 × 10^−07^
*IGF2_pter*	3.3	5.9	1.8	0.002
*LEP*	3.5	7.3	2.1	1.0 × 10^−05^
*CRH*	4.6	7.7	1.7	2.3 × 10^−04^

† Mean within-MZ-pair difference at any locus did not significantly deviate from 0 in both age groups.

### DNA methylation across the complete adult lifespan

#### Changes in within-pair methylation discordance between different age categories

The timing of the occurrence of age-related changes in methylation discordance during adult life was investigated in 219 MZ pairs aged 18–89 years, including an additional 61 middle-aged MZ pairs (30–65 years old) and 25 old MZ pairs (> 65 years old; Table 5). In this extended set of MZ twins, global DNA methylation and methylation at five specific loci, representative of the nine loci studied in the young and old MZ pairs as described before, were measured (Table 1). The observed absolute discordance was plotted for the four stages of adult life: early adulthood (up to 25 years old, *n* = 30 pairs); early middle age (26 years up to 50 years old, *n* = 78 pairs); late middle age (51 years up to 75 years old, *n* = 56 pairs); and old age (over 76 years old, *n* = 54 pairs). Discordance in global DNA methylation was small in all age groups (Fig. 1B). At the loci *IGF2*, *LEP*, and *CRH*, an increase in methylation discordance across the age groups was observed, starting from early adulthood onward at *IGF2* and *CRH* and from middle age onward at *LEP*. Age-related changes in discordance were less apparent at *GNASAS* and *KCNQ1OT1* (Fig. 1B). Plotting the absolute within-pair methylation differences at individual CpG units against age yielded similar results (Figs S1–S6).

To quantify these observed changes, the proportional increase in discordance was also estimated per decade. For global DNA methylation, within-pair discordance was 9% greater each decade (*P* = 3.4 × 10^−06^, Table 4). The greatest increase in within-pair discordance was found at *LEP* with 16% greater discordance each decade (*P* = 2.0 × 10^−08^), and at *IGF2* and *CRH*, within-pair discordance increased each decade with 11% (*P* = 3.8 × 10^−09^) and 8% (*P* = 8.9 × 10^−06^) respectively. At *GNASAS*, discordance did not change significantly with age. At *KCNQ1OT1*, although the discordance was small at all ages, it did increase by 11% each decade (*P* = 0.002; Table 4). These observed relative increases in discordance were confirmed with absolute differences in the amount of discordance when testing the homogeneity of variance in discordance across the age groups at individual CpG units (Table S7).

**Table 4:** Variation of within-pair methylation discordance over the full adult lifespan

	Baseline estimate of variation as SD (% methylation)‡	Increase in variation per decade	
		
Locus†		Proportional increase§ (%)	*P*¶
Global	2.2	9.1	3.4 × 10^−06^
*KCNQ1OT1*	1.9	11.4	0.002
*GNASAS*	3.5	2.7	0.415
*IGF2DMR*	4.4	11.1	3.8 × 10^−09^
*LEP*	4.8	16.0	2.0 × 10^−08^
*CRH*	8.4	8.0	8.9 × 10^−06^

† Loci are ordered from top to bottom as in Fig. 1B from left to right.

‡ The SD of discordance at baseline, estimated from the residual variance and the random effect intercept of the linear mixed model.

§ The proportional (in percentage) increase in variation of discordance each decade, estimated from the random effect of age.

¶ One-sided *P*-value from a *Z*-test on the random effect estimate of age from the same linear mixed model.

Dutch and Danish MZ pairs were investigated in this study. The observation that the increase in within-pair twin discordance was not exclusive to the old age group (predominantly Danish twins) but was already apparent at younger age (Dutch twins) indicated that geographic origin of the MZ pairs did not contribute to our findings. To further exclude the influence of origin, we tested whether age-related changes in methylation variation or discordance were different between old Dutch (*n* = 25 pairs) and Danish (*n* = 67 pairs) twins. No significant influence of country on age-related changes was observed for either variation or discordance (Table S2).

#### The influence of cellular heterogeneity on methylation variation, discordance, and their age-related increases

Methylation was measured on genomic DNA extracted from whole blood, and variation in cellular heterogeneity could induce differences in DNA methylation. In the young twins, neither variation in global DNA methylation (*n* = 132 individuals) nor within-pair differences in global methylation (*n* = 66 pairs) were associated with cellular heterogeneity, approximated by percentage neutrophils, as recently described (Talens *et al.*, 2010). The same was true for the majority of loci (at six of nine loci for DNA methylation and at five of nine loci for within-pair differences; Table S3). The strongest influence of cellular heterogeneity was observed for *LEP* (*P* = 1.2 × 10^−10^ and *P* = 1.0 × 10^−06^ for both tests, respectively). Even in this case, 85% of interindividual variation in *LEP* methylation and 80% of within-pair differences were independent of cellular heterogeneity.

The composition of the leukocyte population changes with age, of which the contribution to our findings was investigated in all Dutch twins. Age-related changes in interindividual variation (*n* = 304 individuals) at three of five loci and in within-pair discordance (*n* = 152 pairs) at four of five loci were not associated with changes in cellular heterogeneity (Table S4). Although an association with cellular heterogeneity was observed (*P* = 0.006 and *P* = 8.2 × 10^−04^ for both tests, respectively), 90% of the age-related changes in either global methylation variation or global methylation discordance were not attributable to it. The strongest influence was observed at *LEP* (*P* = 4.1 × 10^−18^ and *P* = 4.2 × 10^−12^ for both tests, respectively), yet 90% of change in interindividual variation and 85% of change in within-pair discordance were not attributable to cellular heterogeneity. Thus, the age-related changes in DNA methylation observed cannot be explained by changes in leukocyte population composition.

### Longitudinal changes in DNA methylation in old age

Interindividual and within-pair epigenetic variations, global and at the same 5 loci, were also investigated in 19 elderly twin pairs during 10 years of follow-up (DNA samples obtained in 1997 and 2007). Global and locus-specific interindividual methylation variation was modestly larger after 10-year follow-up, except at *CRH* (Table S5). Changes in within-pair methylation discordance showed a similar pattern as observed in comparing changes from young to old adults (Fig. 1: compare C with A and B). Global discordance and that at three loci (*IGF2DMR*, *LEP*, and *CRH*) had increased during the follow-up period, whereas no change was observed at the remaining two loci (*KCNQ1OT1* and *GNASAS*).

### Familial and unique environmental factors

**Fig. 2:**
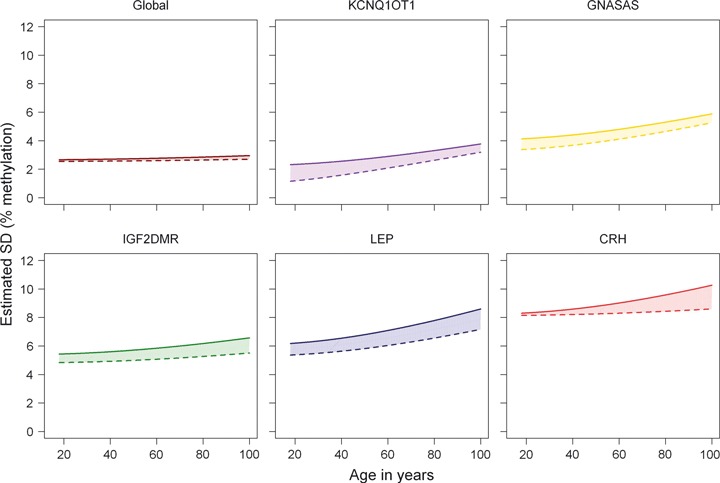
Contribution of familial and unique (individual) environmental factors to increasing methylation variation with age. Estimated changes in the variation in DNA methylation (*y*-axis, given as standard deviation in percent DNA methylation) plotted against the adult lifespan (*x*-axis, age in years). The changes in total variation of DNA methylation (area under the thick line) and the contributions of familial factors (shared environment and genotype, filled-in area between thick and dashed line) and unique factors (individual environment, blank area under the dashed line) were estimated in 219 MZ pairs ranging from 18 to 89 years of age. From top left to bottom right, global DNA methylation and the loci *KCNQ1OT1*, *GNASAS*, *IGF2DMR*, *LEP*, and *CRH* are investigated. Significance of the estimates of total, familial, and individual related increases in variation is given in Table S6 (Supporting information).

The study of MZ twin pairs enables separation of the effects of familial (i.e., genetic and common environment) and unique (individual) environment on the accumulation of DNA methylation differences with age. For global methylation, the total variation was relatively small and mainly attributable to unique environment. The increase in variation with age was limited but significant (*P* = 0.004) and mostly attributable to unique environment (*P* = 0.003; Fig. 2, top left graph; Table S6). The total variation increased significantly with age at all loci in line with the previous analyses (Table S6). This increase could be mainly attributed to unique environmental factors except for the age-related increase in variation at *CRH* methylation which had a familial component (*P* = 0.007).

## Discussion

In this study, we report sustained age-related increases in variation of DNA methylation, in an analysis of 460 individuals, comprising 230 MZ pairs, aged from 18 to 89 years. Previously, this question was investigated using cross-sectional and longitudinal study designs on smaller sample sizes with narrower age ranges (Fraga *et al.*, 2005; Feinberg *et al.*, 2010; Gronniger *et al.*, 2010; Talens *et al.*, 2010). Our study extends their findings over the full adult lifespan, supporting the notion that a gradual accumulation of epigenetic changes, globally and at imprinted and nonimprinted loci, occurs up to very old ages. How such changes affect gene expression remains unclear, although some evidence suggests that small differences in DNA methylation may cause an amplified effect on gene expression (Lillycrop *et al.*, 2008). The increase in epigenetic variation was mainly attributable to unique individual factors that cover both stochastic processes and environmental exposures, between which the design of our study cannot distinguish. This may lead to age-related epigenetic dysregulation and may contribute to the age dependency of common diseases (Jaenisch & Bird, 2003; Bjornsson *et al.*, 2004). However, studies that can appropriately address the latter hypothesis will be complex in their design and execution because of the relatively small effect sizes involved and the tissue- and cell-specific nature of age-related changes (Heijmans & Mill, 2012).

We measured LINE1 methylation to assess global DNA methylation (Wang *et al.*, 2010) and found that the variation in global DNA methylation, both interindividual and within-pair, was small at all ages but increased proportionally with age, in accordance with a longitudinal study over 10 years (Bjornsson *et al.*, 2008). The small amount of global variation observed may relate to the fact that global DNA methylation assays measure methylation at a multitude of similar loci distributed throughout the genome (Bollati *et al.*, 2009; Wang *et al.*, 2010), while the introduction of stochastic changes occurs at individual loci. We observed the most prominent age-related changes in variation at specific candidate loci for common age-related diseases, such as *IGF2*, *LEP*, and *CRH*, of which the expression was shown to be influenced by its DNA methylation (Melzner *et al.*, 2002; Cui *et al.*, 2003; McGill *et al.*, 2006). We observed a substantial difference between the loci in their vulnerability to epigenetic drift, which was consistently found in our various analyses (interindividual variation, within-pair discordance, young versus old individuals, the adult lifespan, or a longitudinal analysis in old subjects). Of interest, the greatest age-related changes were observed for nonimprinted loci, whereas imprinted loci appeared more stable.

MZ twin pairs share characteristics such as age, sex, genotype, and their developmental and childhood environment (e.g., upbringing/education). They may acquire more unique characteristics as they grow older, because different choices on, for instance, lifestyle and occupation can increasingly change their living environments. The shared characteristics, namely their genotype and shared environment, and their individual characteristics, commonly named the unique environment, may both contribute to epigenetic variation (Martin, 2005; Bell & Spector, 2011). We used the MZ twin study design to investigate how much of the age-related increase in variation is contributed by the familial factors and by the unique individual environment (Purcell, 2002; Bell & Spector, 2011). We observed that most of the variation in young adults could be attributed to the individual environment, indicating that DNA methylation is at least partly independent of familial (i.e., genetic) factors. Interestingly, studies on neonate and infant MZ pairs indicated that such differences are already present and indeed increase at a very young age (Ollikainen *et al.*, 2010; Wong *et al.*, 2010). At most loci, we found that the age-related changes in variation were also mostly attributable to the unique individual environment, supporting the idea that age-related changes in DNA methylation may be mostly independent from familial factors (Jaenisch & Bird, 2003; Bjornsson *et al.*, 2004; Martin, 2009). However, at *CRH*, the increase in variation was mostly attributed to familial factors. Residual batch effects seem an unlikely explanation for this observation, considering that the design of this study involved a batch allocation scheme that took age and sex into account and the statistical models contained a factorial variable to adjust for its potential influence. A previous study reported familial clustering of variation over time in global DNA methylation using the LUMA assay (Bjornsson *et al.*, 2008). As we studied MZ twins only, we cannot further distinguish whether this familial component is related to genetic or shared environmental factors, both of which may influence the susceptibility to stochastic or environmentally driven changes in DNA methylation (Sandovici *et al.*, 2003).

In this study, we observed that differences in mean methylation between the young and the old age groups were relatively small or even absent, while increases in variation were generally more substantial. This finding indicates that epigenetic changes accumulating with age are generally nondirectional or are the outcome of many smaller directed changes with, in part, opposite direction. Changes in DNA methylation can be stochastic or environmentally driven; the relative contribution of each source of variation cannot be investigated in our study. Stochastic epigenetic changes can occur without any environmental influence and may be related to imperfect DNA methylation maintenance mechanisms (Petronis, 2006). We observed that the age-related increase in variation of DNA methylation was gradual from early adulthood to old age, which is compatible with stochastic effects (Bjornsson *et al.*, 2004). Environmentally driven epigenetic changes may occur as a consequence of environmental exposures related to, for instance, lifestyle and occupation (Bollati *et al.*, 2007; Gronniger *et al.*, 2010; Breitling *et al.*, 2011). It is even conceivable that stochastic epigenetic changes occur more often under certain environmental conditions, parallel to stochastic genetic mutations that occur after exposure to high UV irradiation.

The power of cross-sectional studies in MZ twins is that large age ranges can be studied in contrast to what is practically possible in longitudinal studies on unrelated individuals. We investigated changes in epigenetic variation, in terms of both interindividual variation and within-pair difference, over a large age range. We observed similar increases in either measure of epigenetic variation throughout the adult lifespan. These results are in line with longitudinal analyses over relatively short time spans in old age, as we report here, middle age (Feinberg *et al.*, 2010; Talens *et al.*, 2010), and childhood (Wong *et al.*, 2010). In view of the consistency of these findings, it is unlikely that generation effects significantly contributed to our findings. Importantly, we were also able to exclude age-related changes in cellular heterogeneity of whole blood as an explanation for our observations, which was previously proposed as a major concern for the interpretation of such studies (Martin, 2005). Whether increased methylation variation in blood has any biological (phenotypic) relevance is an important and as yet unresolved question, although it is likely that other tissues than blood are similarly affected by epigenetic drift (Ronn *et al.*, 2008; Gronniger *et al.*, 2010; Wong *et al.*, 2010). In general, tissues with a high rate of cell division may display more age-related epigenetic variation through stochastic errors in maintaining and transmitting epigenetic information than tissues with lower rates of cell division (Thompson *et al.*, 2010).

In this study, we investigated DNA methylation of MZ twin pairs with ages distributed across the full adult lifespan. In the first phase of the study, we explored differences by comparing young adult twins (18–30 years) with old twins (> 74 years) to generate sufficient contrast between groups. Such age groups at either extreme of the adult lifespan could not be selected from a single twin register. This required a careful consideration of potential biases created by selecting young twins from the Netherlands and old twins from Denmark and additional measurements to confirm the validity of our findings. Genetic differences between the populations were unlikely to play a role, because a genome-wide analysis of SNPs of Northern European countries reported a similar structure of genetic variation in the two countries (McEvoy *et al.*, 2009). Further, similar procedures were used for drawing blood (Christensen *et al.*, 2008; Willemsen *et al.*, 2010), DNA was extracted using standard protocols, and there was no indication for differences in DNA quality [including OD 260/280 measurements, bisulfite (BS) conversion rate and success rate of DNA methylation assays]. Moreover, if DNA quality was different between the populations, one would expect a similar systematic effect on all assays, which could be taken into account in our statistical analysis. In contrast, the loci we studied showed a substantial difference in the degree to which the variation in DNA methylation was higher in old twins, which was absent for *KCNQ1OT1*. More importantly, we experimentally validated the finding from the first phase for six loci. In this second phase, we compared the old Danish twins with a subset of Dutch twins specifically selected for a maximum age overlap [within the limitations of the availability of old twins in the Netherlands twin register (NTR)]. We found no indication for DNA methylation differences between Dutch and Danish twins with a similar old age. Furthermore, the locus-specific associations of DNA methylation variation with age originally observed in the young–old comparison were confirmed both in an investigation of intermediate age ranges selected from the NTR and in a longitudinal investigation of old Danish twins. Taken together, differences in geographic origin or technical variability between populations are unlikely explanations for our observations in phase one of our study, and the second phase yielded further evidence for the occurrence of sustained epigenetic changes during the adult lifespan.

In this study, we demonstrate that epigenetic variation in the population, used as a proxy for stochastic and environmentally driven epigenetic changes in individuals, increases gradually with age up to old age. The rate at which changes are introduced differs between loci and can be considerable at loci regulating transcription of nearby genes. The observed increase was mostly driven by the unique environment. Our results have practical implications for study design in epigenetic studies investigating populations with a large age distribution or a long follow-up time (Foley *et al.*, 2009; Talens *et al.*, 2010). Future research should aim to investigate the relative contribution of stochastic and environmental factors to age-related epigenetic changes and the consequences of these changes for the development of common age-related diseases.

## Experimental procedures

### Study population

The samples in this study are taken from Longitudinal Study of Aging Danish Twins (LSADT) of the Danish Twin Registry (DTR; McGue & Christensen, 2007) and from the Biobank project of the NTR (Willemsen *et al.*, 2010). In the LSADT study, DNA was extracted using the salting out method, and in the Biobank project, QIAamp DNA Blood Maxi (Qiagen, Düsseldorf, Germany) was used. DNA from both sources was of high quality (260/280_BIOBANK_ = 1.80; 260/280_LSADT_ = 1.90).

#### Selection from the Danish Twin Registry (DTR)

The LSADT study, based on the DTR, is a cohort sequential study of elderly Danish twins. LSADT began in 1995 with an assessment of all members of like-sex twin pairs born in Denmark before 1920. The surviving members were followed up every 2 years, and additional cohorts were added at the 1997, 1999, and 2001 assessments and subsequently followed at 2-year intervals. During a home visit in 1997, blood was drawn from 689 individuals, from which DNA was isolated (Skytthe *et al.*, 2006). The LSADT project has been approved by The Danish National Committee on Biomedical Research Ethics (journal VF 20040241). Details on design and data collection were described previously (Christensen *et al.*, 2008).

This study focuses on the monozygotic twin pairs (MZ pairs) of whom DNA was available from the 1997 assessment, (73 years or older; *n* = 108 pairs). To investigate differences in epigenetic variation between young and old MZ pairs, all 36 male MZ pairs and 37 randomly selected female MZ pairs formed a study population named ‘old Danish twins’ (Table 5). For two male MZ pairs and four female MZ pairs, there was insufficient DNA of both co-twins. These MZ pairs were excluded, and the remaining 67 MZ pairs were investigated. For 19 of the LSADT MZ pairs (eight male pairs), a second DNA sample was available from a 10-year follow-up in 2007 for both co-twins. Longitudinal epigenetic changes in the elderly were investigated in these MZ pairs, who were named ‘follow-up Danish twins’ (Table 5).

**Table 5:** Basic characteristics of the different MZ twin pair populations investigated in this study

Population†	*N* (pairs)	No. of male pairs	Age in years, mean (range)	No. of assays studied	Cell counts‡
Young Dutch	66	34	25.2 (18.0–29.8)	10	+
Middle-aged Dutch	61	15	46.3 (30.0–64.0)	6	+
Old Dutch	25	8	70.5 (65.0–78.0)	6	+
Old Danish	67	34	79.3 (74.1–89.0)	10	na
Baseline Danish	19	8	76.6 (73.2–81.8)	6	na
10-year follow-up	19	8	86.5 (83.4–91.8)	6	na
Full adult lifespan**§**	219	91	52.7 (18.0–89.0)	6	

† The designation for the population sample in this study, Dutch twins are participants in the Netherlands Twin Register (NTR), and Danish twins are participants in the Danish Twin Registry (DTR).

‡ Availability of data on the amount of the major leukocyte fractions (neutrophils, lymphocytes, monocytes, basophils, and eosinophils).

§ The population sample covering the full adult lifespan combines the young, middle-aged and old NTR, and the old DTR twins.

#### Selection from the Netherlands Twin Register (NTR)

In 2004, the NTR started a large-scale biological sample collection in twin families to create a resource for genetic studies on health, lifestyle, and personality. Between January 2004 and July 2008, adult participants of the NTR (18 years and above) were invited to the project. During a home visit, fasting blood was drawn, from which DNA was extracted and a hematological profile was obtained, consisting of percentages and numbers of neutrophils, lymphocytes, monocytes, eosinophils, and basophils. The study protocol was approved by Central Ethics Committee on Research Involving Human Subjects of the VU University Medical Center, Amsterdam, an Institutional Review Board certified by the US Office of Human Research Protections (IRB number IRB-2991 under Federal-wide Assurance-3703; IRB/institute codes, NTR 03-180). Details on design, biological sampling, and data collection were described previously (Willemsen *et al.*, 2010).

To investigate differences in epigenetic variation between young and old MZ pairs, 37 MZ pairs of each sex were randomly selected from all NTR MZ pairs who were under 30 years old at sampling (*n* = 98 pairs, 44 male pairs). For three male and five female MZ pairs, there was insufficient DNA of both co-twins. These MZ pairs were excluded, and the remaining 66 MZ pairs were named ‘young Dutch twins’ (Table 5). To investigate differences in epigenetic discordance over the full adult lifespan, 37 MZ pairs were selected from all NTR MZ pairs between 30 and 50 years of age (135 pairs, 34 male pairs) using a block random selection procedure to guarantee an even distribution over the age range. They were combined with all NTR MZ pairs who were above 50 years of age (49 pairs, 16 male pairs). The MZ pairs between 30 and 65 years of age were named ‘middle-aged Dutch twins’ (*n* = 61; 15 male pairs), and the MZ pairs above 65 years of age were named ‘old Dutch twins’ (*n* = 25; eight male pairs; Table 5).

### DNA methylation

#### Assays and measurement

DNA methylation was measured using a quantitatively accurate mass spectrometry–based method (Epityper version 1.05, Sequenom, San Diego, CA, USA; Coolen *et al.*, 2007; Ehrich *et al.*, 2008). A total of 10 DNA methylation assays were measured in this study, the LINE1 assay for global DNA methylation (Wang *et al.*, 2010) and nine assays for DNA methylation at seven specific genomic loci [*IGF2* (3 assays), *LEP*, *CRH*, *ABCA1*, *INS*, *KCNQ1OT1*, and *GNASAS*; Table 1]. Two novel assays were designed to assess methylation of the CpG sites directly telomeric, named *IGF2_pter*, and centromeric, named *IGF2_qter*, of the assay at the *IGF2* locus’ DMR (Heijmans *et al.*, 2007), named *IGF2DMR* for clarity. The primers of each assay were designed to create a PCR bias for completely BS-converted DNA (Li & Dahiya, 2002). More details on the design, features, and measurement of the other eight methylation assays were described in detail previously (Heijmans *et al.*, 2007; Talens *et al.*, 2010; Wang *et al.*, 2010). Briefly, BS conversion of 0.5 μg of genomic DNA using the EZ 96-DNA methylation kit (Zymo Research, Orange, CA, USA) was followed by PCR amplification (primers are given in Table S1A), fragmentation after reverse transcription, and analysis on a mass spectrometer. Fragments that contain one or more CpG sites are called CpG units.

#### Randomization and quality control

All methylation assays were measured in triplicate on the same BS-converted DNA sample. DNA samples of both co-twins of an MZ pair were always allocated to the same batch for BS conversion (on 96-well plate) and PCR amplification (384-well plate, 3 × 124 DNA samples). Each batch contained equal proportions of the age groups measured and the sexes, and each PCR batch contained equal proportions of the BS conversion batches. There were two phases of methylation measurement in this study. First, all ten methylation assays were measured in the young Dutch and the old Danish twins, who were randomly divided over the measurement batches. The ten assays contained a total of 74 measurable CpG units, over which 102 CpG sites were distributed (Table 1). After quality control (Talens *et al.*, 2010), 65 CpG units, containing 93 CpG sites, remained, with a mean call rate of 96.5%.

In the second phase, six methylation assays representing observations on the ten assays were measured in the middle-aged and old Dutch twins, who were randomly divided over the measurement batches, and in the follow-up Danish twins, who were all allocated to a single measurement batch. The six loci contained a total of 47 CpG units, over which 66 CpG sites were distributed. After quality control, 42 CpG units, containing 61 CpG sites, remained both for the Dutch twins (average call rate = 96.4%) and for the follow-up Danish twins (average call rate = 95.7%). These CpG units were the same that passed quality control in the first phase. Table S1B gives the CpG units and CpG sites that passed quality control of each assay and the call rates per assay in both phases.

Bisulfite conversion was assessed using the MassArray R package (Thompson *et al.*, 2009), which identifies CpG-less fragments containing a TpG and a cytosine on the assay's original genomic sequence. It analyzes the mass spectra, treating these fragments as hypothetical CpG sites, because incomplete BS conversion would result in the same mass shift as Cytosine methylation at a CpG site. For both Danish and Dutch twins, this analysis qualified BS conversion as complete within the technical limitation of the method.

### Statistical analysis

#### Definitions

*Methylation variation*: the standard deviation of the mean (SD) of the interindividual differences within a group.

*Within-pair methylation difference*: the within-pair DNA methylation difference at each CpG unit, with DNA methylation of co-twin 1 as the reference: difference = Twin1 − Twin2.

*Methylation discordance*: the range of the within-pair differences in a group. To quantify age-related changes in discordance, the SD of the within-pair differences in a group is used. In figures, the absolute within-pair differences (absolute discordance) are used.

*Days*: a continuous variable for the time between the drawing of blood from each co-twin of a twin pair computed in days, with co-twin 1 as the reference.

*Batch*: a categorical variable with a distinct designation for each combination of PCR and BS batch.

#### Linear mixed models, description of basic models

Linear mixed models were used to test for age-related changes in DNA methylation of the assays, its variation, and its discordance, as previously described (Tobi *et al.*, 2009; Talens *et al.*, 2010). More details on the linear mixed model are given in the Data S1 (Supporting information).

In all the linear mixed models used for testing age-related changes in interindividual methylation variation, DNA methylation was entered as a dependent variable. Individual was the subject variable. Necessary adjustments were made by entering age, sex, twin designation (T1 or T2, to account for nonindependence), batch, and CpG unit as fixed effects. In all the linear mixed models used for testing age-related changes in within-pair methylation discordance, the within-pair difference was entered as a dependent variable. Family was the subject variable. Necessary adjustments were made by entering age_Twin1_, days, sex, batch, and CpG unit as fixed effects. Both basic models were adapted to suit each specific test as described below.

#### Adaptation of basic models for each specific test

DNA methylation, methylation variation, and methylation discordance were compared between young Dutch (*n* = 66 pairs) and old Danish twins (*n* = 67 pairs). Age group (young or old) was added to the models as a random effect to test for differences in variation or discordance and as an extra fixed effect, replacing age, to estimate adjusted group mean methylation or mean within-pair difference and its SD [using the standard error (SE) of the mean] and test for group differences.

Changes in methylation discordance over the full adult lifespan were tested in all Dutch and the old Danish twins (*n* = 219 pairs), and age was entered as a random effect.

Longitudinal methylation variation was investigated in the follow-up Danish twins (*n* = 19 pairs), and the model was adapted as follows: DNA sample (i.e., individual per year of sampling) was the subject variable. Year of sampling (1997 or 2007) was entered as an extra random effect and as an extra fixed effect. Adjustment for age was made using age at first sampling, and no adjustment for batch was required.

Adjusted mean DNA methylation, the differences in means, interindividual variation, and within-pair discordance are all expressed as percentage DNA methylation. The fold change in methylation variation and discordance between groups is expressed as a proportion by dividing the SD in the older group with the SD in the younger group (SD_older_/SD_younger_). The change in discordance over the adult lifespan is expressed as the proportional increase each decade as percentage of the discordance of the previous decade. Significance of the age-related changes in variation or discordance was tested with a one-sided *Z*-test applied on the random effect estimate of age group, age, or sampling time divided by its SE, which adds up to a Wald test.

#### Adaptation of basic models for subsidiary tests

To test whether age effects were similar between Dutch and Danish individuals, methylation variation and discordance were compared between old Dutch twins (*n* = 25 pairs) and old Danish twins (*n* = 67 pairs). Age was entered as an extra random effect. An interaction term age*country was entered as an extra fixed effect, insignificance of which would establish that Dutch and Danes represent the same population.

Nested linear mixed models were used to investigate confounding by leukocyte population heterogeneity, approximated by percentage neutrophils, as recently described (Talens *et al.*, 2010). Confounding of methylation variation and discordance was tested on the young Dutch twins (*n* = 66 pairs). Confounding of age-related changes was tested on all Dutch twins (*n* = 152 pairs). The basic models are as described above, with age also entered as a random effect when testing age-related changes. Nested models had percentage neutrophils for testing its influence on methylation variation and the within-pair difference in percentage neutrophils (Twin 1 − Twin 2) for testing its influence on methylation discordance, added to their corresponding basic model as an extra fixed effect. The amount of confounding is determined by the change in residual variance, or the change in the random effect estimate of age, in the nested model with respect to the basic, as described previously (Talens *et al.*, 2010).

#### Variance component models for twin analysis

In this study, interindividual methylation variation and within-pair methylation discordance have been investigated separately. In the statistical models commonly used in twin research, both aspects of variation can also be investigated simultaneously, thus correcting each component for the other. The classical twin model for MZ twins (Purcell, 2002) postulates that the methylation values (*y*) at a given locus for co-twins 1 and 2 of twin pair *i* are defined by an overall mean (*μ*) that may depend on age, sex, batch, CpG unit, by a random twin-pair effect (*b*_*i*_), the familial environment, which stands for the shared factors of the twin pair, including common environment and genotype, and by a residual error (*e*_*ij*_), the individual environment which stands for the factors that are unique to each co-twin (in formula: *y*_*ij*_ = *μ + b*_*i*_ + *e*_*ij*_). However, this classical twin model is not able to capture that the methylation variance increases with age. We therefore used the following extension of the classical twin model to allow for such age variation: *y*_*ij*_ = *μ* + *b*_*i*_ + *e*_*ij*_ + *age*_*ij*_ × *a*_*i*_ + *age*_*ij*_ × *c*_*ij*_, with *μ*, *b*_*i*_, and *e*_*ij*_ as before and *a*_*i*_ and *c*_*ij*_ quantifying shared (familial) and unique (individual) age effects independently from each other and from *b*_*i*_ and *e*_*ij*_. More details on these MZ twin variance component models (Purcell, 2002) are given in the Data S1 (Supporting information).

In the linear mixed models used to test the individual and familial components of variation in DNA methylation over the adult age range (*n* = 219 pairs), DNA methylation was entered as a dependent variable. Individual and family were both subject variables. Age was entered as a random effect around family (with the intercept) and as a random effect around individual, thereby adjusting each variance component for the other. For necessary adjustments, age, sex, batch, and CpG unit were entered as fixed effects. Significance of the age-related increases in total, familial, and individual variation was tested with a one-sided *Z*-test applied on the random effect estimates of age.

The square root of the resulting random effect estimates represents an estimation of the SD, which, expressed as percentage DNA methylation, is easier to interpret. The total of all variation (residual variance, intercept, and familial and individual age-related estimate) and the individual variation (residual variance and individual age-related estimate) was plotted against age to visualize the familial and individual age-related increase in variation, because total variance minus individual variance represents the familial variance.

## Supporting information

**Data S1** Statistical models.

**Fig. S1** The absolute within MZ twin pair difference in % DNA methylation (*y*-axis) plotted against age (*x*-axis) per CpG unit of the *LEPTIN* locus.

**Fig. S2** The absolute within MZ twin pair difference in % DNA methylation (*y*-axis) plotted against age (*x*-axis) per CpG unit of the *CRH* locus.

**Fig. S3** The absolute within MZ twin pair difference in % DNA methylation (*y*-axis) plotted against age (*x*-axis) per CpG unit of the *IGF2DMR* locus.

**Fig. S4** The absolute within MZ twin pair difference in % DNA methylation (*y*-axis) plotted against age (*x*-axis) per CpG unit of the *KCNQ1OT1* locus.

**Fig. S5** The absolute within MZ twin pair difference in % DNA methylation (*y*-axis) plotted against age (*x*-axis) per CpG unit of the *GNASAS* locus.

**Fig. S6** The absolute within MZ twin pair difference in % global DNA methylation (*y*-axis) plotted against age (*x*-axis) per CpG unit.

**Table S1** (A) primers used for the BS PCR. (B) CpG sites per CpG unit of each assay and assay call rates (CR) after quality control for the two phases of this study.

**Table S2** Significance of the test for interaction between country of origin and the observed age related epigenetic effects.

**Table S3** Influence of percentage neutrophils on methylation variation and discordance in young MZ twins.

**Table S4** Influence of percentage neutrophils on age related changes in methylation variation and discordance.

**Table S5** Mean (SD) DNA methylation and longitudinal change in inter-individual variation in old MZ pairs.

**Table S6** Significance test for the increase in total, familial and individual variation in DNA methylation.

**Table S7** Homogeneity of variance test of the within-pair methylation differences across the age groups per CpG unit.

As a service to our authors and readers, this journal provides supporting information supplied by the authors. Such materials are peer-reviewed and may be re-organized for online delivery, but are not copy-edited or typeset. Technical support issues arising from supporting information (other than missing files) should be addressed to the authors.

## References

[b1] Bell JT, Spector TD (2011). A twin approach to unraveling epigenetics. Trends Genet.

[b2] Bell JT, Pai AA, Pickrell JK, Gaffney DJ, Pique-Regi R, Degner JF, Gilad Y, Pritchard JK (2011). DNA methylation patterns associate with genetic and gene expression variation in HapMap cell lines. Genome Biol.

[b3] Bernstein BE, Meissner A, Lander ES (2007). The mammalian epigenome. Cell.

[b4] Bjornsson HT, Fallin MD, Feinberg AP (2004). An integrated epigenetic and genetic approach to common human disease. Trends Genet.

[b5] Bjornsson HT, Sigurdsson MI, Fallin MD, Irizarry RA, Aspelund T, Cui H, Yu W, Rongione MA, Ekstrom TJ, Harris TB, Launer LJ, Eiriksdottir G, Leppert MF, Sapienza C, Gudnason V, Feinberg AP (2008). Intra-individual change over time in DNA methylation with familial clustering. JAMA.

[b6] Bollati V, Baccarelli A, Hou L, Bonzini M, Fustinoni S, Cavallo D, Byun HM, Jiang J, Marinelli B, Pesatori AC, Bertazzi PA, Yang AS (2007). Changes in DNA methylation patterns in subjects exposed to low-dose benzene. Cancer Res.

[b7] Bollati V, Schwartz J, Wright R, Litonjua A, Tarantini L, Suh H, Sparrow D, Vokonas P, Baccarelli A (2009). Decline in genomic DNA methylation through aging in a cohort of elderly subjects. Mech. Ageing Dev.

[b8] Breitling LP, Yang R, Korn B, Burwinkel B, Brenner H (2011). Tobacco-smoking-related differential DNA methylation: 27K discovery and replication. Am. J. Hum. Genet.

[b9] Cedar H, Bergman Y (2009). Linking DNA methylation and histone modification: patterns and paradigms. Nat. Rev. Genet.

[b10] Christensen K, Bathum L, Christiansen L, Weinstein M, Vaupel JW, Wachter KW (2008). Biological Indicators and Genetic Information in Danish Twin and Oldest-old Surveys. Biosocial Surveys.

[b11] Coolen MW, Statham AL, Gardiner-Garden M, Clark SJ (2007). Genomic profiling of CpG methylation and allelic specificity using quantitative high-throughput mass spectrometry: critical evaluation and improvements. Nucleic Acids Res.

[b12] Cui H, Cruz-Correa M, Giardiello FM, Hutcheon DF, Kafonek DR, Brandenburg S, Wu Y, He X, Powe NR, Feinberg AP (2003). Loss of IGF2 imprinting: a potential marker of colorectal cancer risk. Science.

[b13] Eckhardt F, Lewin J, Cortese R, Rakyan VK, Attwood J, Burger M, Burton J, Cox TV, Davies R, Down TA, Haefliger C, Horton R, Howe K, Jackson DK, Kunde J, Koenig C, Liddle J, Niblett D, Otto T, Pettett R, Seemann S, Thompson C, West T, Rogers J, Olek A, Berlin K, Beck S (2006). DNA methylation profiling of human chromosomes 6, 20 and 22. Nat. Genet.

[b14] Ehrich M, Turner J, Gibbs P, Lipton L, Giovanneti M, Cantor C, van den Boom D (2008). Cytosine methylation profiling of cancer cell lines. Proc. Natl Acad. Sci. USA.

[b15] Feinberg AP, Irizarry RA, Fradin D, Aryee MJ, Murakami P, Aspelund T, Eiriksdottir G, Harris TB, Launer L, Gudnason V, Fallin MD (2010). Personalized epigenomic signatures that are stable over time and covary with body mass index. Sci. Transl. Med.

[b16] Foley DL, Craig JM, Morley R, Olsson CJ, Dwyer T, Smith K, Saffery R (2009). Prospects for epigenetic epidemiology. Am. J. Epidemiol.

[b17] Fraga MF, Ballestar E, Paz MF, Ropero S, Setien F, Ballestar ML, Heine-Suner D, Cigudosa JC, Urioste M, Benitez J, Boix-Chornet M, Sanchez-Aguilera A, Ling C, Carlsson E, Poulsen P, Vaag A, Stephan Z, Spector TD, Wu YZ, Plass C, Esteller M (2005). Epigenetic differences arise during the lifetime of monozygotic twins. Proc. Natl Acad. Sci. USA.

[b18] Gronniger E, Weber B, Heil O, Peters N, Stab F, Wenck H, Korn B, Winnefeld M, Lyko F (2010). Aging and chronic sun exposure cause distinct epigenetic changes in human skin. PLoS Genet.

[b19] Heijmans BT, Mill J (2012). Commentary: the seven plagues of epigenetic epidemiology. Int. J. Epidemiol.

[b20] Heijmans BT, Kremer D, Tobi EW, Boomsma DI, Slagboom PE (2007). Heritable rather than age-related environmental and stochastic factors dominate variation in DNA methylation of the human IGF2/H19 locus. Hum. Mol. Genet.

[b21] Jaenisch R, Bird A (2003). Epigenetic regulation of gene expression: how the genome integrates intrinsic and environmental signals. Nat. Genet.

[b22] Kuroda A, Rauch TA, Todorov I, Ku HT, Al-Abdullah IH, Kandeel F, Mullen Y, Pfeifer GP, Ferreri K (2009). Insulin gene expression is regulated by DNA methylation. PLoS ONE.

[b23] Li LC, Dahiya R (2002). MethPrimer: designing primers for methylation PCRs. Bioinformatics.

[b24] Lillycrop KA, Phillips ES, Torrens C, Hanson MA, Jackson AA, Burdge GC (2008). Feeding pregnant rats a protein-restricted diet persistently alters the methylation of specific cytosines in the hepatic PPAR alpha promoter of the offspring. Br. J. Nutr.

[b25] Martin GM (2005). Epigenetic drift in aging identical twins. Proc. Natl Acad. Sci. USA.

[b26] Martin GM (2009). Epigenetic gambling and epigenetic drift as an antagonistic pleiotropic mechanism of aging. Aging Cell.

[b27] Martin N, Boomsma D, Machin G (1997). A twin-pronged attack on complex traits. Nat. Genet.

[b28] McEvoy BP, Montgomery GW, McRae AF, Ripatti S, Perola M, Spector TD, Cherkas L, Ahmadi KR, Boomsma D, Willemsen G, Hottenga JJ, Pedersen NL, Magnusson PK, Kyvik KO, Christensen K, Kaprio J, Heikkila K, Palotie A, Widen E, Muilu J, Syvanen AC, Liljedahl U, Hardiman O, Cronin S, Peltonen L, Martin NG, Visscher PM (2009). Geographical structure and differential natural selection among North European populations. Genome Res.

[b29] McGill BE, Bundle SF, Yaylaoglu MB, Carson JP, Thaller C, Zoghbi HY (2006). Enhanced anxiety and stress-induced corticosterone release are associated with increased Crh expression in a mouse model of Rett syndrome. Proc. Natl Acad. Sci. USA.

[b30] McGue M, Christensen K (2007). Social activity and healthy aging: a study of aging Danish twins. Twin Res. Hum. Genet.

[b31] Melzner I, Scott V, Dorsch K, Fischer P, Wabitsch M, Bruderlein S, Hasel C, Moller P (2002). Leptin gene expression in human preadipocytes is switched on by maturation-induced demethylation of distinct CpGs in its proximal promoter. J. Biol. Chem.

[b32] Mitsuya K, Meguro M, Lee MP, Katoh M, Schulz TC, Kugoh H, Yoshida MA, Niikawa N, Feinberg AP, Oshimura M (1999). LIT1, an imprinted antisense RNA in the human KvLQT1 locus identified by screening for differentially expressed transcripts using monochromosomal hybrids. Hum. Mol. Genet.

[b33] Ollikainen M, Smith KR, Joo EJ, Ng HK, Andronikos R, Novakovic B, bdul Aziz NK, Carlin JB, Morley R, Saffery R, Craig JM (2010). DNA methylation analysis of multiple tissues from newborn twins reveals both genetic and intrauterine components to variation in the human neonatal epigenome. Hum. Mol. Genet.

[b34] Petronis A (2006). Epigenetics and twins: three variations on the theme. Trends Genet.

[b35] Probst MC, Thumann H, Aslanidis C, Langmann T, Buechler C, Patsch W, Baralle FE, linga-Thie GM, Geisel J, Keller C, Menys VC, Schmitz G (2004). Screening for functional sequence variations and mutations in ABCA1. Atherosclerosis.

[b36] Purcell S (2002). Variance components models for gene-environment interaction in twin analysis. Twin Res.

[b37] Ronn T, Poulsen P, Hansson O, Holmkvist J, Almgren P, Nilsson P, Tuomi T, Isomaa B, Groop L, Vaag A, Ling C (2008). Age influences DNA methylation and gene expression of COX7A1 in human skeletal muscle. Diabetologia.

[b38] Sandovici I, Leppert M, Hawk PR, Suarez A, Linares Y, Sapienza C (2003). Familial aggregation of abnormal methylation of parental alleles at the IGF2/H19 and IGF2R differentially methylated regions. Hum. Mol. Genet.

[b39] Skytthe A, Kyvik K, Bathum L, Holm N, Vaupel JW, Christensen K (2006). The Danish Twin Registry in the new millennium. Twin Res. Hum. Genet.

[b40] Talens RP, Boomsma DI, Tobi EW, Kremer D, Jukema JW, Willemsen G, Putter H, Slagboom PE, Heijmans BT (2010). Variation, patterns, and temporal stability of DNA methylation: considerations for epigenetic epidemiology. FASEB J.

[b41] Thompson RF, Suzuki M, Lau KW, Greally JM (2009). A pipeline for the quantitative analysis of CG dinucleotide methylation using mass spectrometry. Bioinformatics.

[b42] Thompson RF, Atzmon G, Gheorghe C, Liang HQ, Lowes C, Greally JM, Barzilai N (2010). Tissue-specific dysregulation of DNA methylation in aging. Aging Cell.

[b43] Tobi EW, Lumey LH, Talens RP, Kremer D, Putter H, Stein AD, Slagboom PE, Heijmans BT (2009). DNA Methylation differences after exposure to prenatal famine are common and timing- and sex-specific. Hum. Mol. Genet.

[b44] Wang L, Wang F, Guan J, Le J, Wu L, Zou J, Zhao H, Pei L, Zheng X, Zhang T (2010). Relation between hypomethylation of long interspersed nucleotide elements and risk of neural tube defects. Am. J. Clin. Nutr.

[b45] Willemsen G, de Geus EJ, Bartels M, van Beijsterveldt CE, Brooks AI, Estourgie-van Burk GF, Fugman DA, Hoekstra C, Hottenga JJ, Kluft K, Meijer P, Montgomery GW, Rizzu P, Sondervan D, Smit AB, Spijker S, Suchiman HE, Tischfield JA, Lehner T, Slagboom PE, Boomsma DI (2010). The Netherlands Twin Register biobank: a resource for genetic epidemiological studies. Twin Res. Hum. Genet.

[b46] Williamson CM, Turner MD, Ball ST, Nottingham WT, Glenister P, Fray M, Tymowska-Lalanne Z, Plagge A, Powles-Glover N, Kelsey G, Maconochie M, Peters J (2006). Identification of an imprinting control region affecting the expression of all transcripts in the Gnas cluster. Nat. Genet.

[b47] Wong CC, Caspi A, Williams B, Craig IW, Houts R, Ambler A, Moffitt TE, Mill J (2010). A longitudinal study of epigenetic variation in twins. Epigenetics.

