# Supplementary material for: Epigenetic variation during the adult lifespan: cross-sectional and longitudinal data on monozygotic twin pairs
Source: Aging Cell. 2012 Aug;11(4):694–703. doi: 10.1111/j.1474-9726.2012.00835.x (PMC3399918; doi:10.1111/j.1474-9726.2012.00835.x)
Supplement: Supplementary file 1 [file acel0011-0694-SD1.pdf]

Supplementary information on statistical models

Linear mixed models

The linear mixed model uses all available methylation data per locus (assay), i.e. methylation of multiple CpG units per locus, accounts for the correlation between methylation of CpG units within a locus, and using this correlation handles data missing at random. It further enables the inclusion of relevant adjustments on the raw data within the same model. For categorical covariates entered as fixed effect the model estimates adjusted means and tests for differences between groups. This reduces to a t-test, when a single CpG unit with complete methylation data is tested without adjustments. For continuous covariates entered as fixed effect the model estimates and tests the adjusted linear relation between the dependent and covariate. This reduces to regression analysis when a single CpG unit with complete data is tested. For covariates entered as random effect the model estimates the increase in variance of the dependent variable per unit change of the random effect covariate. A Z-test applied on the estimate of variance divided by its standard error (SE), tests the significance of this change in variance of the dependent variable, which in essence adds up to a Wald test.

Variance component analysis in twin studies

Define y_{i1} and y_{i1} to be the methylation values at a given locus for twins 1 and 2 of twin-pair $\boldsymbol{i}$. The classical twin model for MZ twins postulates that $\boldsymbol{y_{ij} = \mu + b_i + e_{ij}}$, where $\boldsymbol{\mu}$ is an overall mean that may depend on age, sex, batch, CpG unit, $\boldsymbol{b_i}$ is a random twin-pair effect, assumed normal with mean zero and variance $\boldsymbol{\sigma^2_b}$, and $\boldsymbol{e_{ij}}$ is the residual error [unique environment], assumed normal with mean zero and variance

σ^2_e, and independent of b_i. The b_i stands for shared familial factors, including common environment and genotype, and the e_{ij} for unique environment. This model implies that the methylation variance equals $\sigma^2_{tot} = \sigma^2_b + \sigma^2_e$, the correlation between the two methylation values within a twin-pair is the intraclass correlation, given by $\rho = \sigma^2_b / \sigma^2_{tot}$. The within-pair difference $y_{i1} - y_{i2}$ is seen to have a normal distribution with mean zero and variance $2\sigma^2_e$. However, this classical twin model is not able to capture 1) increases of the methylation variance with age, and 2) increases of the variance of the within-pair differences with age. We therefore propose the following extension of the classical twin model, similar to the variance components models in twin analysis (Purcell S. 2002, Twin Res 5 (6):554-571), to allow for such age related variation: $y_{ij} = \mu + b_i + e_{ij} + age_{ij} * a_i + age_{ij} * c_{ij}$, with μ, b_i and e_{ij} as before, and a_i and c_{ij} mean zero normal random variables with variances σ^2_a and σ^2_c, independent from each other and from b_i and e_{ij}. The a_i and c_{ij} quantify shared (family) and unique (individual) age effects, respectively. For this extended twin model, the methylation variance equals $\sigma^2_{tot} = \sigma^2_b + \sigma^2_e + age^2 * (\sigma^2_a + \sigma^2_c)$, and when the ages of the two twins in the twin-pair are approximately equal, then the correlation between the two methylation values within a twin-pair is given by $\rho = (\sigma^2_b + age^2 * \sigma^2_a) / \sigma^2_{tot}$. In that case, the within-pair difference $y_{i1} - y_{i2}$ for this extended model has a normal distribution with mean zero and variance $2(\sigma^2_e + age^2 * \sigma^2_c)$. So for the extended model, both total variance, and the variance of the within-pair differences increase quadratically with age.

Supplementary Figures

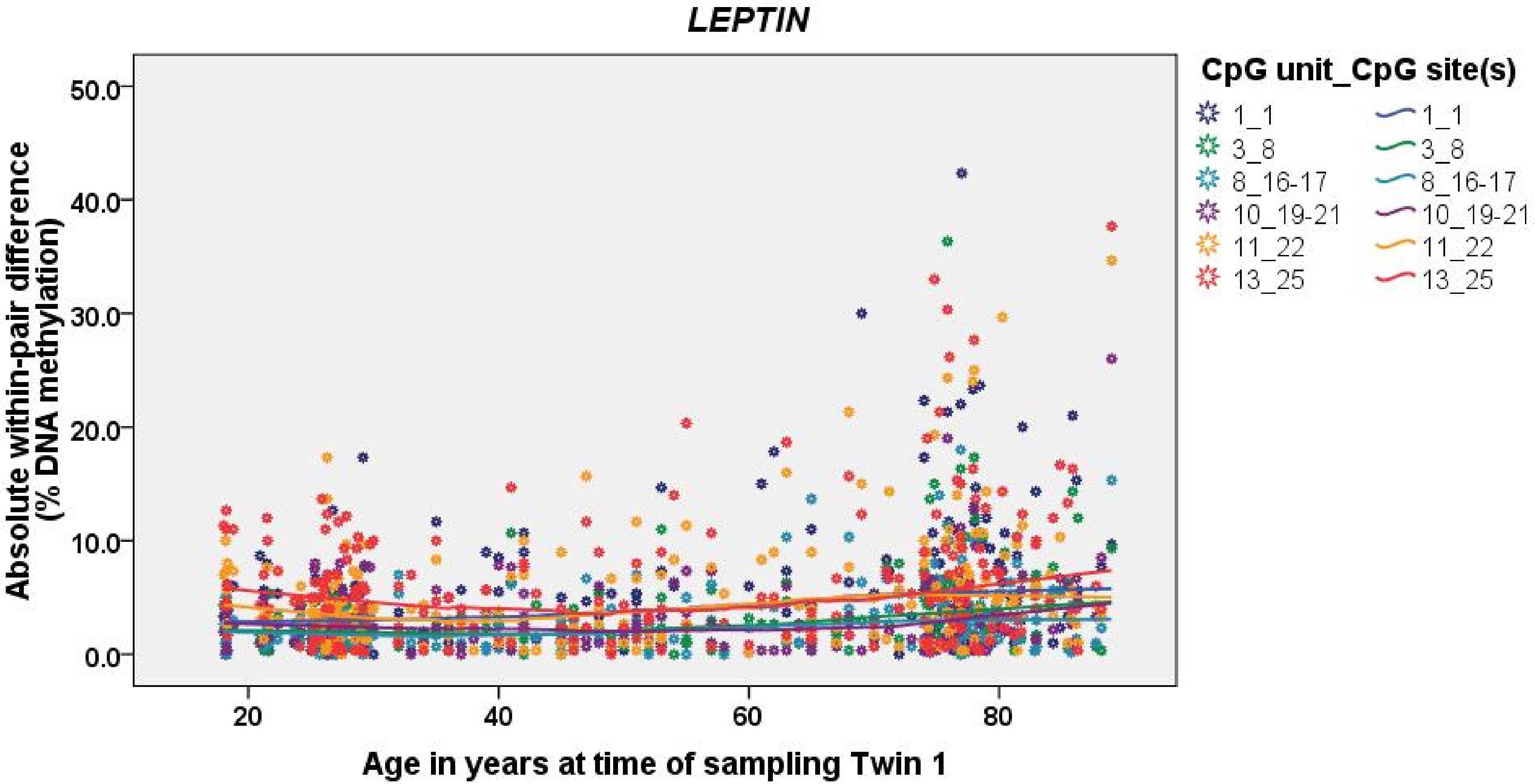

Supplementary Figure S1: The Absolute within MZ twin pair difference in % DNA methylation (y-axis) plotted against age (x-axis) per CpG unit of the *LEPTIN* locus. Each dot represents the absolute within pair difference of one MZ pair at one CpG unit. The different CpG units are represented by different colours. The lines are a loess lines that show the average trend for the change in methylation discordance at a CpG site with age.

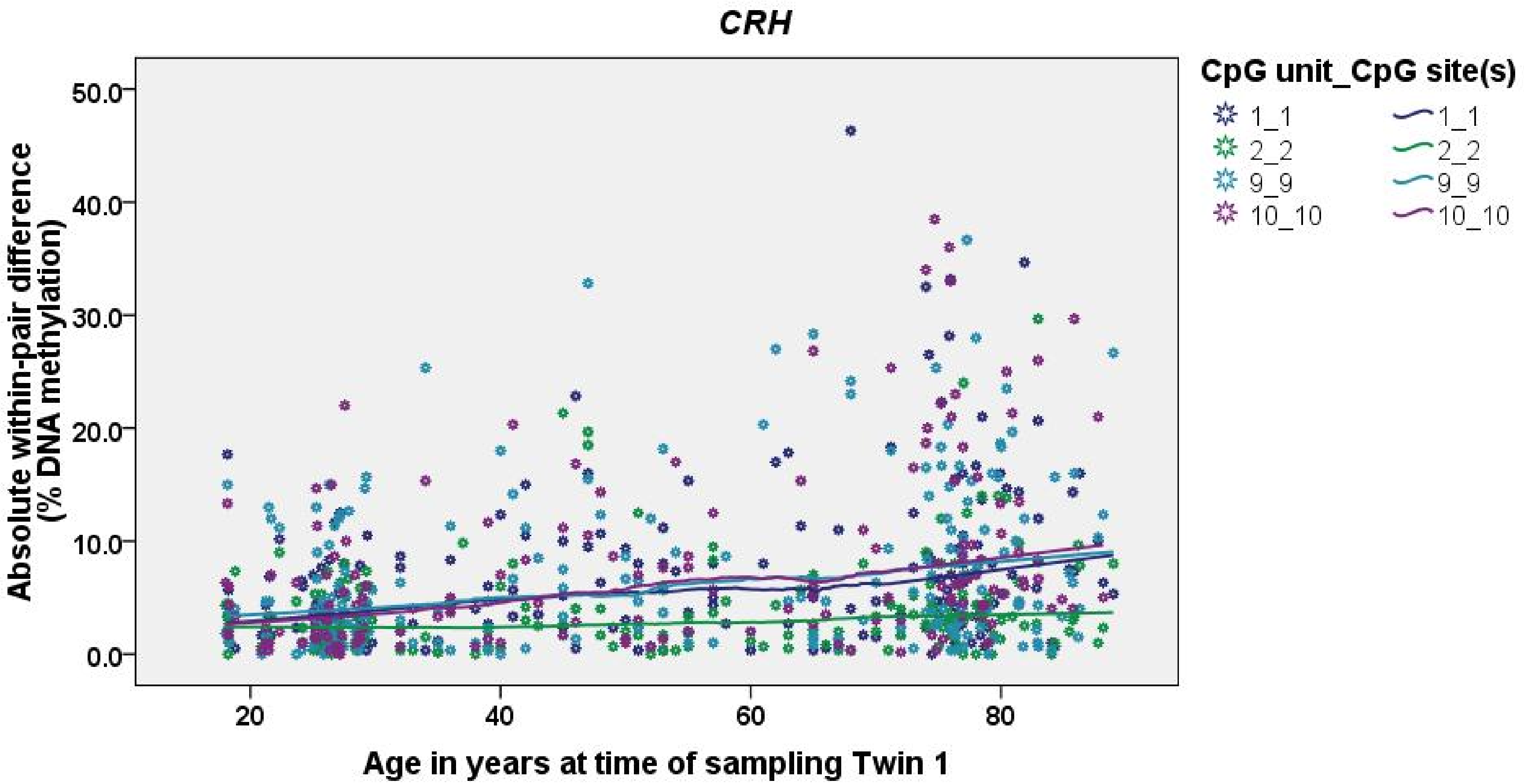

Supplementary Figure S2: The Absolute within MZ twin pair difference in % DNA methylation (y-axis) plotted against age (x-axis) per CpG unit of the *CRH* locus. Each dot represents the absolute within pair difference of one MZ pair at one CpG unit. The different CpG units are represented by different colours. The lines are a loess lines that show the average trend for the change in methylation discordance at a CpG site with age.

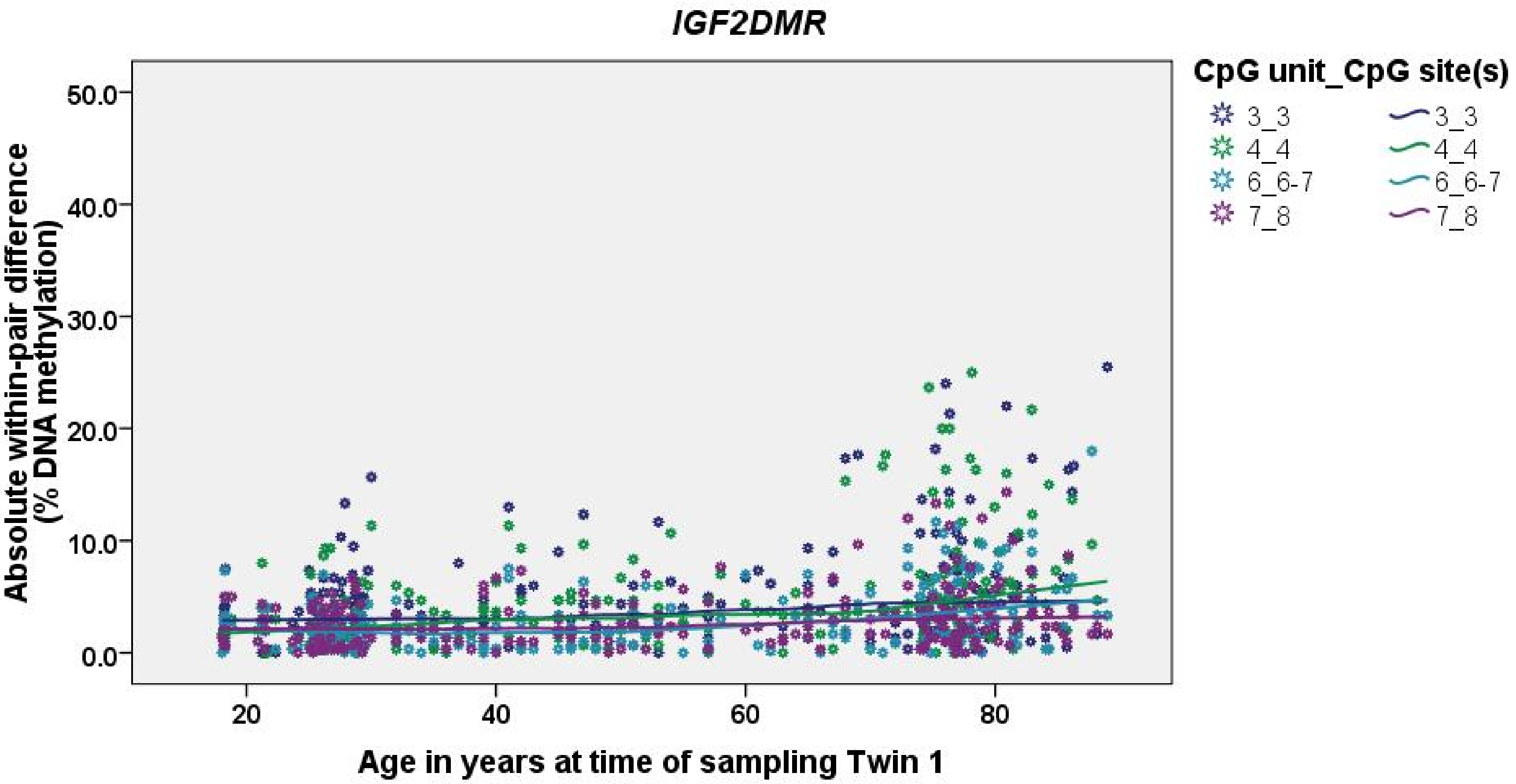

Supplementary Figure S3: The Absolute within MZ twin pair difference in % DNA methylation (y-axis) plotted against age (x-axis) per CpG unit of the *IGF2DMR* locus. Each dot represents the absolute within pair difference of one MZ pair at one CpG unit. The different CpG units are represented by different colours. The lines are a loess lines that show the average trend for the change in methylation discordance at a CpG site with age.

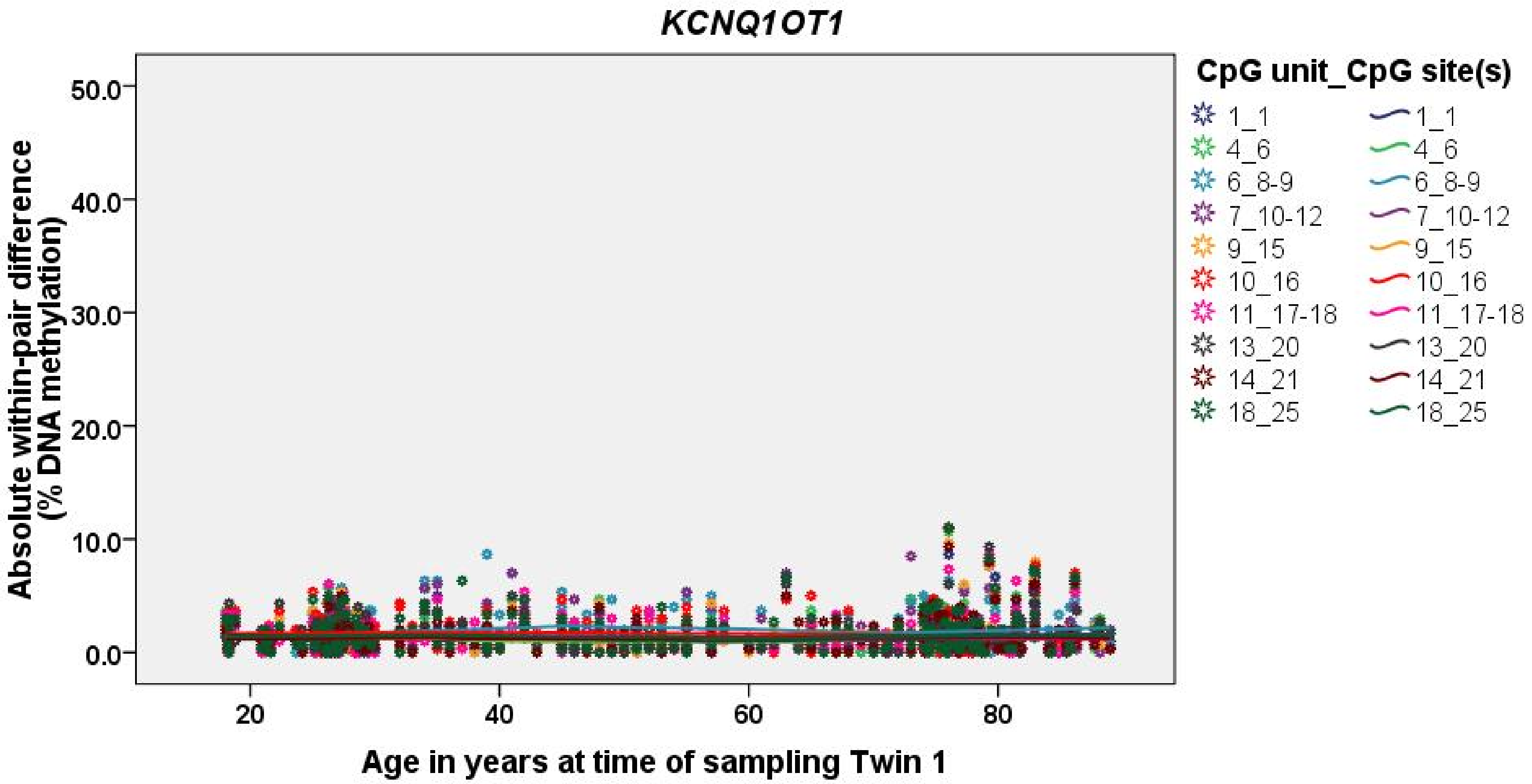

Supplementary Figure S4: The Absolute within MZ twin pair difference in % DNA methylation (y-axis) plotted against age (x-axis) per CpG unit of the *KCNQ1OT1* locus. Each dot represents the absolute within pair difference of one MZ pair at one CpG unit. The different CpG units are represented by different colours. The lines are a loess lines that show the average trend for the change in methylation discordance at a CpG site with age.

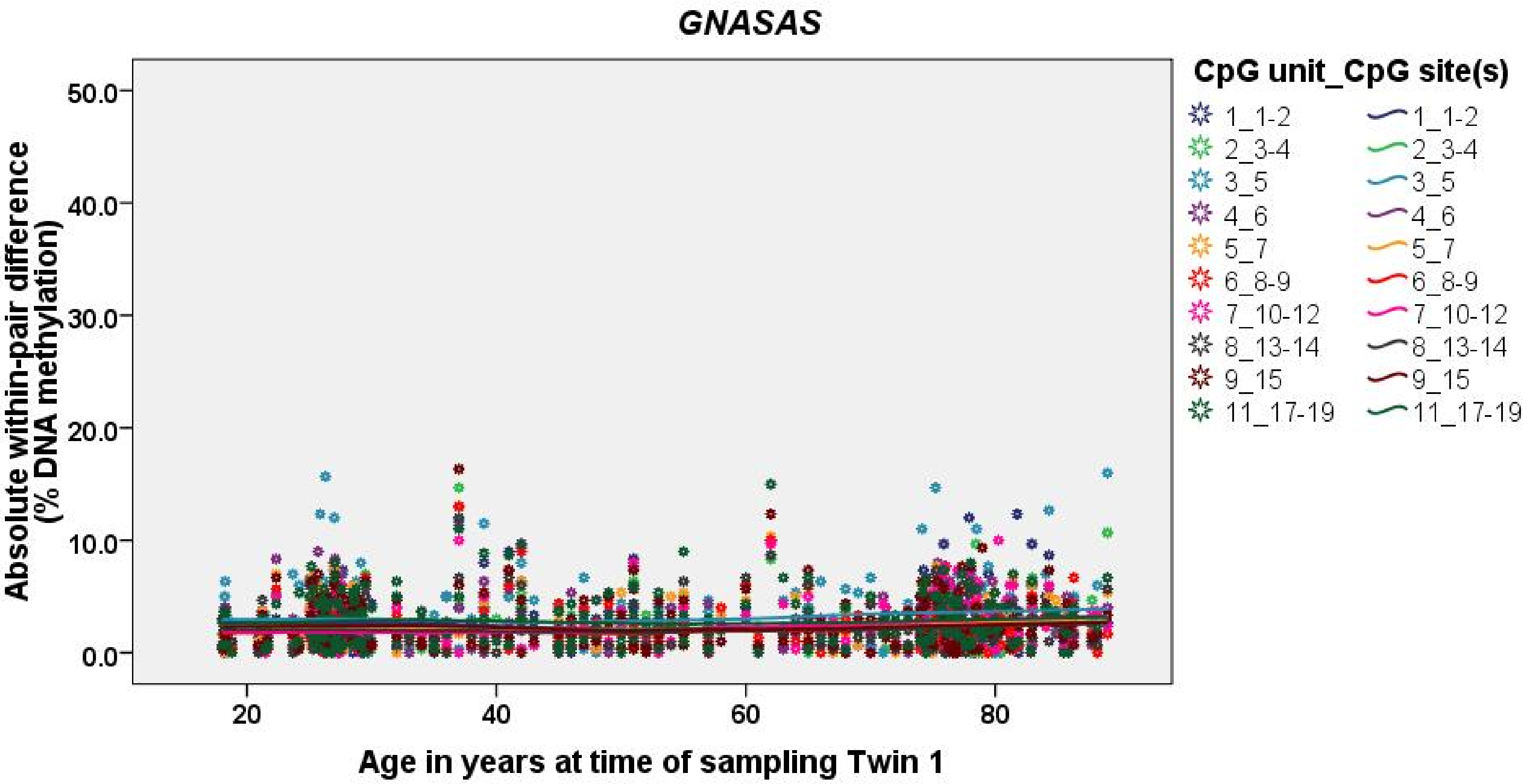

Supplementary Figure S5: The Absolute within MZ twin pair difference in % DNA methylation (y-axis) plotted against age (x-axis) per CpG unit of the *GNASAS* locus. Each dot represents the absolute within pair difference of one MZ pair at one CpG unit. The different CpG units are represented by different colours. The lines are a loess lines that show the average trend for the change in methylation discordance at a CpG site with age.

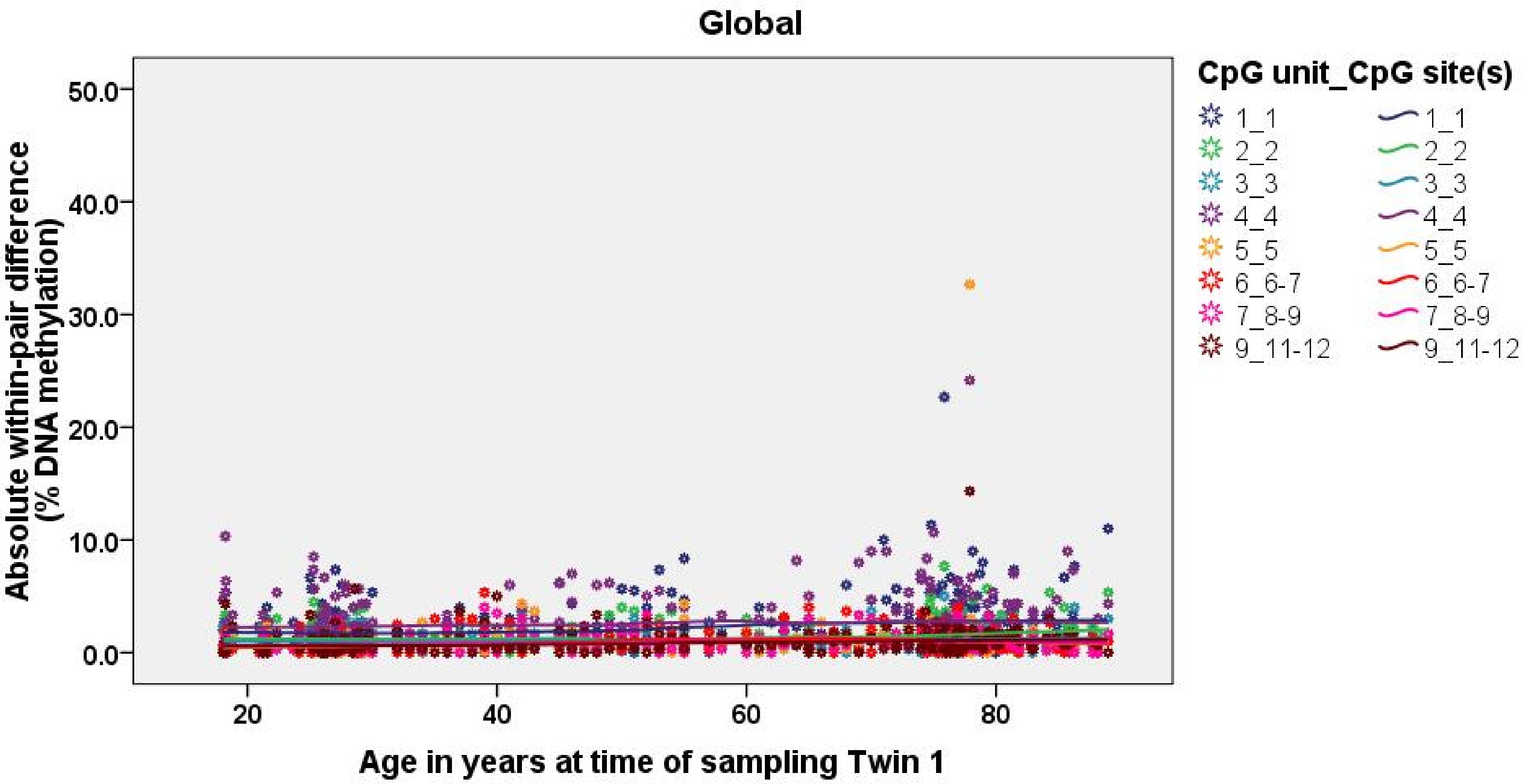

Supplementary Figure S6: The Absolute within MZ twin pair difference in % global DNA methylation (y-axis) plotted against age (x-axis) per CpG unit. Each dot represents the absolute within pair difference of one MZ pair at one CpG unit. The different CpG units are represented by different colours. The lines are a loess lines that show the average trend for the change in methylation discordance at a CpG site with age.

Supplementary tables

Table S1A: primers used for the BS PCR.

Locus (assay)[a]	Forward primer	Reverse primer
ABCA1	ATTTTATTGGTGTTTTTGGTTGT	ATCAAAACCTATACTCTCCCTCCTC
CRH	TGGTTGTTGTTTTTTTGGTAGG	AATTTCTCCACTCCAAAACCTAAA
GNASAS	GTAATTTGTGGTATGAGGAAGAGTGA	TAAATAACCCAACTAAATCCCAACA
IGF2_pter	GTTGTGTGTTTAGTGGTTTTTGTTG	AAAAAATTTACCTAAAAAAAACTTCCC
IGF2_DMR	TGGATAGGAGATTGAGGAGAAA	AAACCCCAACAAAAACCACT
IGF2_qter	GATGAGGTTTTTTTATTTGTAGGGG	AAAACCAAAATCCTAACAACTACCC
INS	GTTTTGAGGAAGAGGTGTTGA	ACCTAAAATCCAACCACCCTAA
KCNQ1OT1	TTTGGTAGGATTTTGTTGAGGAGTTTT	CTCACACCCAACCAATACCTCATAC
LEP	GTTTTTGGAGGGATATTAAGGATTT	CTACCAAAAAAAACCAACAAAAAAA
LINE1	GTGTGAGGTGTTAGTGTGTTTTGTT	ATATCCCACACCTAACTCAAAAAAT
10-mer tag	AGGAAGAGAG + primer	
T7-tag		CAGTAATACGACTCACTATAGGGAGAAGGCT + primer

Table S1B: CpG sites per CpG unit of each assay and assay call rates (CR) after quality control for the two phases of this study.

	Young Dutch and old Danish MZ pairs		Middle-aged and old Dutch MZ pairs		Follow-up Danish MZ pairs	
Locus (assay)[a]	CpG sites analyzed[b]	CR (%)	CpG sites analyzed[b]	CR (%)	CpG sites analyzed[b]	CR (%)
ABCA1[c]	**1[c]**, 2*, **3-4[c]**, **5[c]**, **6-9[c]**, **15-16, 17-18, 19-21, 24, 25**	99.1[c]	NA		NA	
CRH	**1, 2,** 3*, 4*, 8*, **9, 10**	93.4	Same CpG sites	89.7	Same CpG sites	88.5
GNASAS	**1-2, 3-4, 5, 6, 7, 8-9, 10-12, 13-14, 15, 17-19**	97.4	Same CpG sites	98.2	Same CpG sites	98.9
IGF2_pter	**1, 2, 3, 4, 5**	99.2	NA		NA	
IGF2_DMR	**3, 4, 6-7, 8**	99.7	Same CpG sites	99.1	Same CpG sites	99.3
IGF2_qter	1*, **2,** 7*, **8, 9,** 11*, **12-13**	76.5	NA		NA	
INS	**2, 3, 4, 5, 6**	98.9	NA		NA	
KCNQ1OT1	**1,** 2*, **6, 8-9, 10-12, 15, 16, 17-18, 20, 21, 25**	100	Same CpG sites	98.7	Same CpG sites	98.7
LEP	**1, 8, 16-17, 19-21, 22, 25,** 28*	98.2	Same CpG sites	95.7	Same CpG sites	94.1
LINE1	**1, 2, 3, 4, 5, 6-7, 8-9, 11-12**	99.5	Same CpG sites	96.9	Same CpG sites	94.6

a: Loci are ordered alphabetically in both tables

b: CpG site number is counted from the forward primer onward, CpGs that passed quality control are given in bold, CpGs that failed are marked by an asterix (*)

c: The average call rate of the 4 methylated CPG units at the 5' end of the amplicon (underlined) is given, the call rate for all CpG units was 99.5 %

Table S2: Significance of the test for interaction between country of origin and the observed age related epigenetic effects

	$P_{interaction}$[b]	
**Locus (assay)**[a]	**Inter- individual variation**	**Within-pair discordance**
Global	.739	.401
KCNQ1OT1	.444	.929
GNASAS	.182	.163
IGF2DMR	.465	.739
LEP	.098	.423
CRH	.285	.385

a: Loci are ordered from top to bottom as in Figure 1B from left to right

b: The test was done on a population of the old Dutch MZ pairs and old Danish MZ pairs

Table S3: Influence of percentage neutrophils on methylation variation and discordance in young MZ twins

Locus (assay)[a]	Inter-individual variation in DNA methylation		Within-pair methylation discordance	
	% Variation explained[b]	p-value[c]	% Discordance explained[d]	p-value[c]
Global	0.2	.464	1.0	.101
KCNQ1OT1	0.1	.299	0.8	.519
GNASAS	0.2	.227	1.3	.164
ABCA1	0.1	.365	11.4	$3.7 * 10^{-04}$
INS	0.6	.112	0.4	.157
IGF2DMR	0.3	.546	4.8	.008
IGF2_qter	2.8	.002	3.0	.045
IGF2_pter	1.9	.029	1.8	.062
LEP	13.7	$1.2 * 10^{-10}$	19.4	$1.0 * 10^{-06}$
CRH	0.3	.546	0.6	.762

a: Loci are ordered from top to bottom as in Figure 1A from left to right

b: Percentage of variation attributable to percentage neutrophil assessed from change in residual variance

c: Two sided p-value for the effect of neutrophil percentage in the appropriate nested linear mixed model

d: Percentage of twin discordance attributable to neutrophil percentage assessed from change in residual variance

Table S4: Influence of percentage neutrophils on age related changes in methylation variation and discordance

	Inter-individual variation in DNA methylation		Within-pair methylation discordance	
**Locus (assay)[a]**	**% Age related variation[b]**	**p-value[c]**	**% Age related discordance[d]**	**p-value[c]**
Global	10.0	.006	8.7	$8.2 * 10^{-04}$
KCNQ1OT1	0.0	.935	0.0	.794
GNASAS	1.2	.048	0.6	.578
IGF2DMR	3.8	.149	5.1	.132
LEP	9.7	$4.1 * 10^{-18}$	15.1	$4.2 * 10^{-12}$
CRH	0.5	.462	0.5	.631

a: Loci are ordered from top to bottom as in Figure 1B from left to right

b: Percentage of age related variation attributable to neutrophil percentage assessed from change in random effect estimate of age

c: Two sided p-value for the effect of neutrophil percentage in the appropriate nested linear mixed model

d: Percentage of age related twin discordance attributable to neutrophil discordance assessed from change in random effect estimate of age

Table S5: Mean (SD) DNA methylation and longitudinal change in inter-individual variation in old MZ pairs

	**Mean (SD) in % DNA methylation** [b]			**Inter-individual variation**	
**Locus**[a]	**Baseline**	**10 year follow-up**	$\mathbf{P}_{\mathbf{Mean}}$ [c]	**Proportional increase**[d]	$\mathbf{P}_{\mathbf{variation}}$ [e]
Global	59.3 (2.9)	62.0 (3.0)	.003	3.7 %	.400
KCNQ1OT1	30.1 (4.6)	31.7 (4.9)	.271	4.6 %	.999
GNASAS	40.7 (3.2)	42.6 (3.2)	.056	2.6 %	.442
IGF2DMR	54.2 (9.8)	46.0 (10.2)	.008	4.1 %	.301
LEP	31.5 (13.8)	34.0 (14.8)	.575	7.2 %	.046
CRH	67.3 (12.4)	68.2 (12.4)	.827	0.4 %	.344

a: Loci are ordered from top to bottom as in Figure 1C from left to right

b: DNA methylation and SD are adjusted for correlation between the CpG units, family relations, age, and sex

c: Two-sided p-value for testing of group means with a linear mixed model accounting for CpG units, sex, age, and family relations

d: The increase in SD during the follow-up period is given in proportion (percentage) to the SD at baseline

e: One-sided p-value for testing group variation with a Z-test on the estimates of variance from the same linear mixed model

Table S6: Significance test for the increase in total, familial and individual variation in DNA methylation

**Locus**[a]	**Total variation**[b]	**Familial variation**[b]	**Individual variation**[b]
Global	.004	.077	.003
KCNQ1OT1	$1.8 * 10^{-15}$	.999	$1.8 * 10^{-15}$
GNASAS	$1.2 * 10^{-05}$	.378	$1.5 * 10^{-11}$
IGF2DMR	.002	.077	$3.2 * 10^{-04}$
LEP	$3.5 * 10^{-05}$	.071	$4.2 * 10^{-10}$
CRH	$8.7 * 10^{-04}$	.007	.055

a: Loci are ordered from top to bottom as in Figure 2 from top left to bottom right

b: One-sided p-value for testing age related increase in variation with a Z-test on the corresponding estimates of variance from the same linear mixed model, effect sizes are shown in Figure2

Table S7: Homogeneity of variance test for the within-pair methylation differences across the age groups per CpG unit

		Up to 25 years old[a]			26 to 50 years old[a]			50 to 75 years old[a]			Over 75 years old[a]			
**Locus_CpG unit**	**CpG site(s)**	**SD[b]**	**Min[b]**	**Max[b]**	**SD[b]**	**Min[b]**	**Max[b]**	**SD[b]**	**Min[b]**	**Max[b]**	**SD[b]**	**Min[b]**	**Max[b]**	**$P_{Levene's}$[c]**
Global_1	1	2.8	-6.7	5.7	2.5	-6.2	7.3	5.0	-11.3	22.7	4.1	-9.0	11.0	.003
Global_2	2	1.8	-3.7	4.5	1.6	-4.0	4.0	2.2	-5.0	7.7	2.4	-5.7	5.3	.024
Global_3	3	1.2	-2.3	2.3	1.3	-3.3	2.7	1.6	-3.7	5.0	1.6	-4.0	3.7	.119
Global_4	4	3.9	-5.7	10.3	3.2	-6.0	7.0	4.2	-9.0	10.7	4.7	-7.0	24.2	.546
Global_5	5	1.1	-3.3	2.3	1.2	-2.7	4.3	1.6	-3.0	4.3	4.6	-3.2	32.7	.190
Global_6	6-7	1.0	-2.3	2.0	1.3	-5.3	3.0	1.8	-4.0	3.7	1.4	-3.7	4.0	.006
Global_7	8-9	0.8	-1.0	1.7	1.2	-4.0	2.3	1.5	-3.3	3.3	1.3	-3.3	3.0	.048
Global_9	11-12	1.3	-1.7	4.3	1.4	-5.7	2.7	1.4	-2.7	3.7	2.3	-14.3	3.0	.386
KCNQ1OT1_1	1	1.6	-3.3	3.3	2.1	-5.0	4.7	2.0	-6.7	4.7	2.8	-7.7	8.7	.057
KCNQ1OT1_4	6	1.9	-3.7	3.7	2.0	-5.7	5.7	2.0	-5.0	4.7	2.8	-8.3	10.7	.439
KCNQ1OT1_6	8-9	1.7	-3.0	3.7	3.0	-8.7	6.0	2.6	-6.3	5.0	2.8	-4.7	7.3	.072
KCNQ1OT1_7	10-12	1.5	-2.3	2.7	2.4	-7.0	5.3	2.4	-8.5	4.7	2.9	-5.7	11.0	.099
KCNQ1OT1_9	15	1.5	-2.3	3.7	1.9	-4.7	5.3	2.0	-5.0	4.3	3.0	-7.7	9.7	.048
KCNQ1OT1_10	16	2.2	-3.3	5.3	2.2	-4.7	5.0	2.2	-5.0	4.7	3.1	-8.0	11.0	.717
KCNQ1OT1_11	17-18	1.6	-3.3	3.3	2.2	-6.0	5.3	1.8	-5.0	3.7	2.6	-8.7	7.3	.240
KCNQ1OT1_13	20	1.8	-4.3	4.3	2.0	-5.0	4.7	1.8	-6.0	3.3	2.5	-9.3	6.0	.549
KCNQ1OT1_14	21	1.3	-2.3	2.7	1.7	-4.0	5.0	1.9	-5.0	4.3	2.8	-8.0	9.3	.023
KCNQ1OT1_18	25	1.8	-3.0	4.7	2.2	-6.3	4.7	2.1	-6.7	4.3	3.1	-8.3	11.0	.089

a: Population sizes: ≤ 25 yrs, n = 30 pairs; 26 – 50 yrs, n = 78 pairs; 51 – 75 yrs, n = 56 pairs; ≥ 76 yrs, n = 54 pairs

b: Standard deviation of within pair difference (SD) minimum (Min) and maximum (Max) differences are given in % DNA methylation

c: Two-sided p-value from Levene’s test for homogeneity of variances across the age groups

Table S7 continued

Locus_CpG unit	CpG site(s)	Up to 25 years old[a] SD[b]	Min[b]	Max[b]	26 to 50 years old[a] SD[b]	Min[b]	Max[b]	50 to 75 years old[a] SD[b]	Min[b]	Max[b]	Over 75 years old[a] SD[b]	Min[b]	Max[b]	$P_{Levene's}$[c]
GNASAS_1	1-2	3.0	-6.0	4.7	4.0	-13.0	7.3	3.3	-10.0	9.7	4.1	-12.3	9.7	.399
GNASAS_2	3-4	2.6	-5.3	5.0	3.3	-14.7	6.0	3.1	-8.3	6.7	3.8	-9.7	10.7	.280
GNASAS_3	5	4.3	-7.0	12.3	4.8	-15.7	7.0	4.5	-14.7	7.7	5.7	-16.0	7.7	.357
GNASAS_4	6	3.8	-8.3	9.0	3.5	-11.3	7.0	3.1	-10.0	6.7	3.6	-6.3	7.3	.357
GNASAS_5	7	3.2	-7.0	6.0	3.5	-13.0	6.3	3.4	-10.3	7.7	3.4	-6.7	6.0	.927
GNASAS_6	8-9	2.6	-6.0	6.0	3.2	-13.0	5.3	3.0	-10.0	6.0	3.2	-6.3	7.7	.507
GNASAS_7	10-12	2.6	-6.0	6.0	3.3	-10.0	6.0	3.3	-9.7	7.0	3.8	-10.0	6.3	.146
GNASAS_8	13-14	2.5	-6.0	6.3	3.3	-12.0	6.7	3.1	-8.7	7.3	2.9	-6.0	5.7	.455
GNASAS_9	15	3.2	-6.7	7.0	3.7	-16.3	6.0	3.6	-12.3	7.3	3.4	-7.7	9.3	.910
GNASAS_11	17-19	3.0	-7.7	5.7	4.1	-11.0	8.0	4.1	-15.0	7.3	3.7	-7.0	8.0	.335
IGF2DMR_3	3	3.5	-7.5	5.0	5.0	-15.7	12.3	6.6	-18.2	10.7	9.6	-24.0	25.5	$6.0*10^{-05}$
IGF2DMR_4	4	2.9	-8.0	7.0	4.4	-11.3	9.7	7.1	-23.7	16.7	9.5	-25.0	21.7	$1.6*10^{-07}$
IGF2DMR_6	6-7	2.9	-7.3	5.0	2.6	-7.0	7.5	4.1	-10.7	11.7	5.9	-18.0	10.7	$3.3*10^{-07}$
IGF2DMR_7	8	2.6	-5.0	4.3	3.0	-6.7	7.3	4.3	-12.0	13.3	5.1	-14.3	12.0	$9.6*10^{-04}$
LEP_1	1	4.0	-8.0	8.7	4.9	-17.3	11.7	9.1	-22.3	30.0	10.6	-23.3	42.3	$6.8*10^{-05}$
LEP_3	8	2.8	-5.0	5.3	2.7	-5.0	10.7	7.0	-36.3	15.0	6.5	-17.3	16.3	$7.3*10^{-06}$
LEP_8	16-17	2.5	-5.0	5.0	2.7	-7.0	7.0	4.9	-11.3	14.0	5.3	-15.3	18.0	$4.9*10^{-04}$
LEP_10	19-21	3.7	-8.0	6.3	3.4	-8.0	7.7	4.3	-19.0	7.3	6.4	-26.0	11.2	.005
LEP_11	22	4.9	-8.0	10.0	5.3	-15.7	17.3	8.2	-24.3	21.3	10.1	-34.7	29.7	.002
LEP_13	25	6.8	-12.0	13.7	6.0	-12.3	14.7	10.2	-30.3	33.0	11.0	-37.7	16.3	.008
CRH_1	1	5.5	-7.7	17.7	6.8	-12.5	22.8	13.1	-46.3	32.5	10.1	-16.0	34.7	.003
CRH_2	2	3.5	-7.3	9.0	5.6	-19.7	21.3	4.5	-9.5	12.0	8.0	-29.7	14.0	.012
CRH_9	9	6.4	-12.0	15.0	8.6	-32.8	25.3	11.7	-27.0	28.3	12.2	-26.7	36.7	.015
CRH_10	10	5.7	-11.3	14.7	7.5	-22.0	20.3	15.3	-36.0	38.5	12.4	-29.7	26.0	$2.4*10^{-04}$
